# Effects of domestic violence on menopausal symptoms, sexual function, and quality of life: a cross-sectional study

**DOI:** 10.61622/rbgo/2025rbgo16

**Published:** 2025-04-30

**Authors:** Laís Lima Ferreira, Charles Francisco Ferreira, Maria Celeste Osório Wender

**Affiliations:** 1 Universidade Federal do Rio Grande do Sul Hospital de Clínicas de Porto Alegre Porto Alegre RS Brazil Hospital de Clínicas de Porto Alegre, Universidade Federal do Rio Grande do Sul, Porto Alegre, RS, Brazil.; 2 Universidade Federal do Rio Grande do Sul Instituto de Ciências Básicas da Saúde Porto Alegre RS Brazil Instituto de Ciências Básicas da Saúde, Universidade Federal do Rio Grande do Sul, Porto Alegre, RS, Brazil.

**Keywords:** Climacteric, Postmenopause, Menopause, Quality of life, Domestic violence, Violence against women, Anxiety, Sexuality, Surveys and questionnaires

## Abstract

**Objective:**

To investigate the association between lifetime experience of domestic violence and climacteric symptoms, sexual function, and quality of life in climacteric women in Rio Grande do Sul, Brazil.

**Methods:**

A cross-sectional study was conducted with 700 pre-, peri-, and postmenopausal women, recruited online via an anonymous questionnaire (REDCap platform). Women aged 40 to 65 years, residing in Rio Grande do Sul, and classified by the STRAW+10 criteria were included. Climacteric symptoms and sexual function were assessed using the 10-item Cervantes Scale (CS-10) and the 6-item Female Sexual Function Index (FSFI-6). Data were analyzed using SPSS version 18.0; quantitative data as median [IQR], qualitative as frequencies. Group comparisons used Kruskal-Wallis, Chi-Square, and Spearman's correlation between violence against women (VAW) and/or climacteric groups on CS-10 or FSFI-6. Significance was set at 5%.

**Results:**

The median [IQR] age of pre- (46 [43 - 50] years), peri- (50 [47 – 52] years), and postmenopausal (55 [51 – 58] years) were different among groups. Prevalence rates of psychological (38.8%), sexual (34.9%), and physical (21.3%) violence were observed. Postmenopausal women showed the poorest outcomes. Premenopausal women experiencing violence had severe anxiety, while postmenopausal women reported feeling worthless. Various sexual dysfunctions were associated with violence, including low desire, lubrication issues, and sexual pain.

**Conclusions:**

Domestic violence was linked to worse climacteric symptoms, sexual function, and quality of life, particularly in postmenopausal women. These findings underscore the need for improved care and public policies to enhance safety and well-being among women of all ages.

## Introduction

The menopausal transition marks a significant period of change in women's lives.^(1)^ These changes encompass various aspects: vasomotor symptoms, characterized by hot flashes and night sweats; physical changes, including alterations in body composition and the atrophy of sexual and urinary organs; behavioral shifts, such as sleep disturbances, mood fluctuations, and psychiatric disorders; cognitive alterations, such as memory loss and difficulties with concentration; and increased susceptibility to certain chronic diseases, such as cardiovascular diseases and osteoporosis.^(2)^

The onset of menopause typically occurs around the age of 46 to 50 years in women, making a natural physiological process characterized by the gradual reduction, and ultimately cessation, of estrogen production in the ovaries.^(3-5)^ While a considerable body of literature links hypoestrogenism to symptoms of the climacteric/menopausal transition, this relationship remains inadequately elucidated, with limited research exploring the potential influence of women's life experiences on climacteric symptoms.^(5,6)^

Studies examining the ramifications of experiencing violence have revealed an association between such experiences and a heightened intensity of climacteric symptoms and dissatisfaction with sexual life, poorer sleep quality, psychiatric illnesses, a higher prevalence of risky behaviors, chronic illnesses and subclinical cardiovascular diseases.^(7-12)^

It is a well-established fact that one in three women has experienced physical and/or sexual violence at some point in their lives, often perpetrated by their partners.^(13)^ Furthermore, it is alarming that the Americas rank as the second continent with the highest rate of femicides.^(14)^ In Brazil, women aged between 45 and 64 years represent approximately 19% of feminicide cases.^(15)^ Despite the existence of some published studies, these indices remain both underestimated and outdated, highlighting the need for further investigation.^(16,17)^ Thus, this study aims to investigate the association between experiences of domestic violence (psychological, sexual, and physical) and climacteric symptoms, sexual function, and quality of life among women aged 40 to 65 residing in southern Brazil. Additionally, it seeks to analyze how these experiences vary according to the menopausal stage, with a focus on adverse health outcomes in postmenopausal women.

## Methods

This prospective cross-sectional study collected data through an online questionnaire administered between March and October 2023. Women aged 40 to 65, residing in the state of Rio Grande do Sul, Brazil, who had or had not experienced domestic violence, were included.

Potential participants in the study were invited to participate through the distribution of the online questionnaire link, which was shared on social media, on the Hospital de Clínicas de Porto Alegre (HCPA) website, in local newspapers, and through materials such as flyers and posters. The link directed women to an initial page containing information about the study and instructions for completing the questionnaire. This included a video demonstrating what participants would encounter in the questionnaire once they agreed to participate, starting with the acceptance of the informed consent form and explaining how the questions were presented, how responses were saved, and how participants could complete the questionnaire in one sitting or in two parts, with the second part to be completed within 7 days.

Participants were classified into three groups based on the stages of the Stages of Reproductive Aging Workshop +10 (STRAW) classification: 1) premenopausal women (stages -3a, -3b); 2), perimenopausal women (stages -2, -1), and 3) postmenopausal women (stages +1a, +1b, +1c, +2).^(18)^ The classification was performed using the standard criteria for menstrual cycle alterations to determine menopausal status. Specifically, premenopausal status was defined by regular menstrual cycles (< 60 days of amenorrhea), perimenopausal status was characterized by irregular menstrual cycles with a history of changes in cycle length or flow (≥ 60 days of amenorrhea), and postmenopausal status was confirmed by the absence of menstruation for at least 12 consecutive months. Exclusion criteria included individuals outside the specified age range, absence of data regarding menstrual irregularities or menopausal status, non-natural menopause, transgender individuals (both male and female), and failure to provide email contact. Participants were not restricted based on their use of hormonal treatment.

Sample size calculations were performed using Programs for Epidemiologists for Windows (WinPEPI) version 11.63 software, based on proportions described by Lawrenz et al. for the lifetime victims of violence aged 40 to 59 (31.5%).^(19)^ With a 95% confidence interval, 5% significance level, and accounting for a 15% potential loss to follow-up, minimum of 474 climacteric women were deemed necessary for the study.

Data for the study were collected and managed using Research Electronic Data Capture (REDCap) tools hosted at HCPA. The collected data, both qualitative and quantitative, pertained to the participant characteristics, health status, climacteric symptoms and quality of life, female sexual function, and experiences of domestic and family violence.

To assess climacteric symptoms and quality of life, the 10-item Cervantes Scale (CS-10) was used.^(20)^ This Likert scale questionnaire comprises ten questions that evaluate four domains: health and menopause, sexuality, couple relationship and psychological aspects. Responses are scored from 0 to 5, with 0 indicating "no symptoms" and 5 indicating "very severe" symptoms. Higher overall scores indicate greater severity of the climacteric symptoms. Female sexual function was evaluated using the 6-item Female Sexual Function Index (FSFI-6), validated for use in middle-aged women.^(21)^ This Likert scale assessed desire, arousal, lubrication, orgasm, satisfaction, and pain, with higher scores indicating better sexual function.

To assess domestic violence, the World Health Organization (WHO) experienced violence against women (VAW) questionnaire was used.^(22)^ This instrument evaluates physical, sexual, and psychological violence perpetrated by a partner over the last 12 months or longer periods.

All instruments used were presented in Portuguese to the participants and have been previously validated for Brazilian Portuguese applicable to climacteric women.^(20-22)^

To minimize participant burden, the questionnaire provided the option of completing it in one sitting or over two sessions, with the latter allowing responses within 7 days after initial access. Participants received automated email reminders to complete both parts of the questionnaire. They also received information about domestic violence, including types of violence against women and resources available under the Maria da Penha law. Additionally, participants had access to support from the researchers and the women's collective "Vozes Inquietas" (Unheard Voices), which offers voluntary services in psychology, lar, social assistance, and police support, either free of charge or at a nominal cost for necessary cases.

Data processing, including database double entry, review, and analysis was conducted using Statistical Package for the Social Sciences (SPSS), version 18.0. [SPSS Inc. Released 2009. PASW Statistics for Windows, Version 18.0. Chicago: SPSS Inc.]. Quantitative data were expressed as median and interquartile range ([IQR], 25^th^ – 75^th^ percentiles), based on the Shapiro-Wilk test. Qualitative variables were described using absolute (n) and relative (%) frequencies. Comparisons among the pre-, peri-, and postmenopausal groups, as well as between groups of women reporting no violence (NV) and those reporting any violence (V), were performed using the Kruskal-Wallis test with Dunn post hoc analysis (KW) or Chi-Square test with adjusted residual analysis (χ²), respectively.

Spearman's rho (ρ) coefficients were calculated to determine correlations between CS-10 or FSFI-6 total scores and variables related to violence against women (VAW). The level of significance was set at 5% for all analyses.

This study received approval from the Institutional Research Ethics Committee 5.334.856 (CAAE - *Certificado de Apresentação de Apreciação Ética*: 56123622.3.0000.5327).

## Results

**Figure 1:**
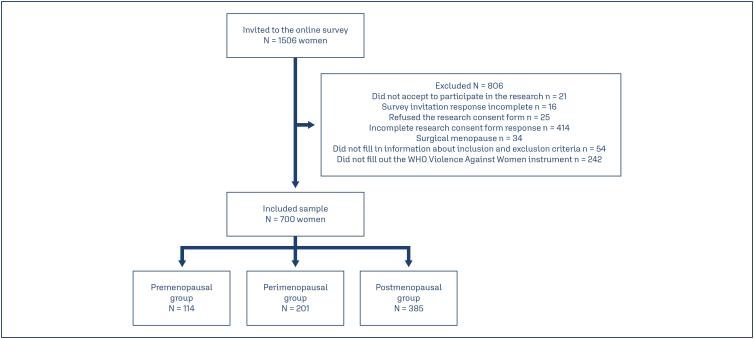
Participant selection process

The participant selection flowchart is shown in figure 1. A total of 1,506 women accessed the questionnaire. Of these, some did not accept or did not complete the following instruments: the form to participate in the research (refused n = 21; incomplete information n = 16), the consent form to participate in the research (refused n = 25, incomplete information n = 414), the eligibility screening instrument (incomplete information n = 54; surgical menopause n = 34), and the WHO VAW (incomplete information n = 242). Therefore, the final sample consisted of 700 women who were categorized according to the STRAW+10 criteria as pre- (n = 114, 16.3%), peri- (n = 201, 28.7%), or post-menopause (n = 385, 55.0%).

n – absolute frequency

The sociodemographic and health status characterization of the participants is presented in table 1. In general, most participants were white (82.3%), heterosexual (95.0%), with more than three monthly minimum wages (in R$, 60.4%) and works registered (59.0%). The median [IQR] ages of pre- (46 [43 - 50]), peri- (50 [47 – 52]), and postmenopausal (55 [51 – 58]) were significantly different (KW, p ≤ 0.0001). In addition, postmenopausal group was associated with a higher frequency of divorced (17.7%) and windowed (3.1%) women (χ² = 0.040), medical consultation for menopausal transition symptoms (79.5%, χ² ≤ 0.001), and chronic diseases (42.3%, χ² = 0.048), mainly type 2 diabetes (8.1%, χ² = 0.002), hypercholesterolemia (9.6%, χ² = 0.019), and back pain (11.7%, χ² = 0.032). No significant differences were observed in median [IQR] body mass index (BMI, in kg/m²) among the groups (KW = 0.339). In special, some participants of the premenopausal group reported being overweight since adulthood (26.3%), while postmenopausal women reported being overweight after menopause (34.8%) (χ² ≤ 0.001).

**Table 1:** Sociodemographic and health status characterization

Variables		Total (n = 700)	Premenopausal (n = 114)	Perimenopausal (n = 201)	Postmenopausal (n = 385)	*p-value
Color/Ethnicity						0.272
	White	576(82.3)	91(79.8)	173(86.1)	312(81.0)	
	Black	52(7.4)	11(9.6)	11(5.5)	30(7.8)	
	Brown	67(9.6)	10(8.8)	16(8.0)	41(10.6)	
	Yellow	2(0.3)	0(0.0)	1(0.5)	1(0.3)	
	Other	3(0.4)	2(1.8)	0(0.0)	1(0.3)	
Marital status						**0.040**
	Single/without partner	119(17.0)	16(14.0)	41(20.4)	62(16.1)	
	Single/with partner	127(18.1)	24(21.1)	37(18.4)	66(17.1)	
	Married	337(48.1)	64(56.1)	96(47.8)	**177(46.0)**	
	Divorced	103(14.7)	10(8.8)	25(12.4)	**68(17.7)**	
	Widow	14(2.0)	0(0.0)	2(1.0)	12(3.1)	
Age ^#^		52.00 [48.00 – 56.00]	46.00 [43.00 – 50.00]*	50.00 [47.00 – 52.00]**	55.00 [51.00 – 58.00]^***^	≤ **0.0001**
Sexual orientation						0.795
	Heterosexual	665(95.0)	107(93.9)	190(94.5)	368(95.6)	
	Homosexual (Lesbian)	11(1.6)	3(2.6)	4(2.0)	4(1.0)	
	Bisexual	17(2.4)	3(2.6)	6(3.0)	8(2.1)	
	Other	7(1.0)	1(0.9)	1(0.5)	5(1.3)	
Education (years)		17.00 [13.00 – 20.00]	17.00 [12.00 – 20.00]	18.00 [14.00 – 20.00]	16.00 [13.00 – 20.00]	0.324
Monthly minimum wage						0.506
	Up to half	13(1.9)	4(3.5)	2(1.0)	7(1.8)	
	Half to one	37(5.3)	7(6.1)	7(3.5)	23(6.0)	
	One to two	96(13.7)	17(14.9)	22(10.9)	57(14.8)	
	Two to three	131(18.7)	20(17.5)	43(21.4)	68(17.7)	
	More than three	423(60.4)	66(57.9)	127(63.2)	230(59.7)	
Works registered						0.143
	No	287(41.0)	51(44.7)	71(35.3)	165(42.9)	
	Yes	413(59.0)	63(55.3)	130(64.7)	220(57.1)	
Body weight (kg)		72.00 [64.00 – 84.00]	74.00 [68.00 – 88.00]*	75.00 [65.00 – 87.00]*	70.00 [62.00 – 80.00]**	**0.005**
Height (m)		1.63 [1.58 – 1.68]	1.64 [1.60 – 1.68]*	1.64 [1.59 – 1.68]*	1.61 [1.58 – 1.66]**	**0.001**
Body mass index (kg/m²)		27.30 [24.12 – 31.66]	27.62 [24.65 – 32.24]	27.59 [24.07 – 32.32]	27.03 [24.00 – 31.22]	0.339
Have children						0.425
	No	126(18.0)	18(15.8)	42(20.9)	66(17.1)	
	Yes	574(82.0)	96(84.2)	159(79.1)	319(82.9)	
Number of children		1.00 [0.00 – 1.00]	1.00 [0.00 – 1.00]	1.00[0.00 – 1.00]	1.00 [0.00 – 1.00]	0.998
Premature children						0.925
	No	475(82.8)	80(83.3)	130(81.8)	265(83.1)	
	Yes	99(17.2)	16(16.7)	29 (18.2)	54(16.9)	
Last menstruation age		49.00 [45.00 – 52.00]	NA	NA	49.00 [45.00 – 52.00]	NA
Medical consultation for symptoms						≤ 0.001
	No	200(28.6)	**59(51.8)**	62(30.8)	79(20.5)	
	Yes	500(71.4)	55(48.2)	139(69.2)	**306(79.5)**	
Treatment for symptoms						**0.027**
	No	492(70.3)	**92(80.7)**	139(69.2)	261(67.8)	
	Yes	208(29.7)	22(19.3)	62(30.8)	124(32.2)	
Body weight variation						0.734
	No	201(28.7)	36(31.6)	58(28.9)	107(27.8)	
	Yes	499(71.3)	78(68.4)	143(71.1)	278(72.2)	
Overweight						≤ **0.001**
	Not overweight	213(30.4)	36(31.6)	64(31.8)	113(29.4)	
	Since childhood	45(6.4)	8(7.0)	17(8.5)	20(5.2)	
	Since adolescence	32(4.6)	4(3.5)	8(4.0)	20(5.2)	
	Since adulthood	123(17.6)	**30(26.3)**	35(17.4)	58(15.1)	
	Since menopause	185(26.4)	13(11.4)	38(18.9)	**134(34.8)**	
	Other	102(14.6)	23(20.2)	**39(19.4)**	40(10.4)	
Infertility						0.098
	No	630(90.0)	106(93.0)	186(92.5)	338(87.8)	
	Yes	70(10.0)	8(7.0)	15(7.5)	47(12.2)	
HIV+						0.745
	No	697(99.6)	114(100.0)	200(99.5)	383(99.5)	
	Yes	3(0.4)	0(0.0)	1(0.5)	2(0.5)	
Chronic disease						**0.048**
	No	428(61.1)	**80(70.2)**	126(62.7)	222(57.7)	
	Yes	272(38.9)	34(29.8)	75(37.3)	**163(42.3)**	
Which chronic disease						
Arterial hypertension		138(19.7)	12(10.5)	40(19.9)	86(22.3)	0.021
	Type 1 diabetes	2(0.3)	0(0.0)	0(0.0)	2(0.5)	0.440
	Type 2 diabetes	38(5.4)	1(0.9)	6(3.0)	**31(8.1)**	**0.002**
	Hypercholesterolemia	50(7.1)	4(3.5)	9(4.5)	**37(9.6)**	**0.019**
	Heart diseases	9(1.3)	0(0.0)	2(1.0)	7(1.8)	0.290
	Stroke	3(0.4)	0(0.0)	1(0.5)	2(0.5)	0.745
	Arthritis or rheumatism	31(4.4)	3(2.6)	9(4.5)	19(4.9)	0.576
	Back pain	64(9.1)	8(7.0)	11(5.5)	**45(11.7)**	**0.032**
	Anxiety	98(14.0)	12(10.5)	30(14.9)	56(14.5)	0.501
	Depression	79(11.3)	12(10.5)	24(11.9)	43(11.2)	0.925
	Other mental disorder	16(2.3)	6(5.3)	3(1.5)	7(1.8)	0.065
	Asthma or asthmatic bronchitis	49(7.0)	8(7.0)	19(9.5)	22(5.7)	0.242
	Lung diseases	8(1.1)	3(2.6)	1(0.5)	4(1.0)	0.222
	Chronic kidney failure	2(0.3)	0(0.0)	1(0.5)	1(0.3)	0.722
	Other chronic diseases	86(12.3)	12(10.5)	26(12.9)	48(12.5)	0.811

Data presented as absolute (n) and relative (%) frequencies, or median [md] and interquartile range ([IQR], percentiles 25^th^ and 75^th^). Minimum wage = R$ 1.320,00; U$ = 268.68. Legend: NA – not applicable. HIV – human immunodeficiency virus. kg – kilogram. m – meter. p – statistical significance index.

*/**/*** Indicate differences among groups. *Chi-Squared test with adjusted residual analysis or Kruskal-Wallis test with Dunn *post hoc*, when applicable. Significance set at 5% for all analysis.

# Total N = 698 (99.7%)

Participants’ lifestyle and habits characterization is displayed in table 2. The postmenopausal group had a higher frequency of participants who reported "never drinking" (34.5%), while women of the perimenopausal group reported alcohol intake 1 to 2 times a week (43.8%) (χ² = 0.001). In general, most participants were non-smokers (68.0%), did not report consuming illicit drugs in the last year (95.0%), and consume coffee (89.1%) 1 to 2 times a day (53.0%).

**Table 2:** Lifestyle and habits characterization

Variables		Total (n = 700)	Premenopausal (n = 114)	Perimenopausal (n = 201)	Postmenopausal (n = 385)	*p-value
Smoking						0.307
	No	476(68.0)	85(74.6)	140(69.7)	251(65.2)	
	Yes	68(9.7)	11(9.6)	19(9.5)	38(9.9)	
	Ex-smoker	156(22.3)	18(15.8)	42(20.9)	96(24.9)	
Cigarettes per day						0.920
	Less than 5	16(23.5)	4(36.4)	5(26.3)	7(18.4)	
	5 to 10	20(29.4)	3(27.3)	5(26.3)	12(31.6)	
	11 to 20	26(38.2)	3(27.3)	7(36.8)	16(42.1)	
	More than 20	6(8.8)	1(9.1)	2(10.5)	3(7.9)	
Illicit drug (last year)						0.471
	No	665(95.0)	108(94.7)	188(93.5)	369(95.8)	
	Yes	35(5.0)	6(5.3)	13(6.5)	16(4.2)	
Illicit drug (last year)						
	Cocaine	4(0.6)	0(0.0)	3(1.5)	1(0.3)	0.116
	Aspirated Ritalin	1(0.1)	0(0.0)	0(0.0)	1(0.3)	0.664
	Unprescribed tranquilizers	10(1.4)	1(0.9)	4(2.0)	5(1.3)	0.690
	Marijuana, Hash or Skank	28(4.0)	5(4.4)	11(5.5)	12(3.1)	0.375
Alcohol intake						**0.001**
	Never drink	205(29.3)	29(25.4)	43(21.4)	**133(34.5)**	
	1 to 2 times a month	254(36.3)	45(39.5)	67(33.3)	142(36.9)	
	1 to 2 times a week	223(31.9)	35(30.7)	**88(43.8)**	100(26.0)	
	Once a day	16(2.3)	4(3.5)	3(1.5)	9(2.3)	
	More than once a day	2(0.3)	1(0.9)	0 (0.0)	1(0.3)	
Coffee intake						0.438
	No	76(10.9)	11(9.6)	18(9.0)	47(12.2)	
	Yes	624(89.1)	103(90.4)	183(91.0)	338(87.8)	
Coffee intake (daily)						0.629
	Occasionally	55(8.8)	10(9.7)	13(7.1)	32(9.5)	
	1 to 2 coffees	330(53.0)	51(49.5)	95(51.9)	184(54.6)	
	3 to 4 coffees	196(31.5)	35(34.0)	58(31.7)	103(30.6)	
	More than 5 coffees	42(6.7)	7(6.8)	17(9.3)	18(5.3)	

Data presented as absolute (n) and relative (%) frequencies; p – statistical significance index. Numbers highlighted in bold indicate association between categories.

* Chi-Squared test with adjusted residual analysis. Significance set at 5% for all analysis

The CS-10 data is shown in table 3. Regarding hot flashes and/or night sweats, the postmenopausal group was associated with ‘very severe’ symptoms (16.4%), the perimenopausal group was associated with "severe" symptom (23.9%), and the premenopausal group was associated with "not presenting" this symptom (34.2%) (χ² = 0.007). Analyzing vaginal discomfort and/or dryness, both the pre- (43.0%) and the perimenopausal (45.3%) groups were associated with "no symptom", while the postmenopausal group had "very severe" discomfort/dryness (23.4%) (χ² ≤ 0.001). As we have noticed in some studies carried out by our research group, a higher proportion of "very severe" skin dryness was found associated with the postmenopausal group (29.9%), while "no skin dryness" was associated with the premenopausal group (χ² ≤ 0.001). On the other hand, most postmenopausal women were not associated with urine leak symptom (65.7%), while both, the peri- (17.9%) and the premenopausal (14.9%) groups, were (χ² = 0.001). In fact, the median [IQR] of vaginal discomfort and/or dryness and skin dryness in postmenopausal women was higher compared to other groups (2 [0 – 4] and 3 [2 – 5], respectively; KW ≤ 0.001 for both). In addition, the median [IQR] of hot flashes and/or night sweats was higher in the perimenopausal (3 [1 – 4]) and the postmenopausal (2 [1 – 4]) groups, in relation to the premenopausal group (1 [0 – 3]) (KW ≤ 0.001). Postmenopausal women showed lower median [IQR] of feeling anxious or nervous (3 [1 – 4]) in relation to the premenopausal women (3 [2 – 5]) (KW = 0.024).

**Table 3:** 10-item Cervantes Scale (CS-10)

Variable		Total (n = 700)	Premenopausal (n = 114)	Perimenopausal (n = 201)	Postmenopausal (n = 385)	*p-value
Hot flashes and/or night sweats						**0.007**
	0 (No symptom)	171(24.4)	**39(34.2)**	45(22.4)	87(22.6)	
	1	108(15.4)	22(19.3)	31(15.4)	55(14.3)	
	2	95(13.6)	16(14.0)	22(10.9)	57(14.8)	
	3	109(15.6)	17(14.9)	31(15.4)	61(15.8)	
	4	125(17.9)	15(13.2)	**48(23.9)**	62(16.1)	
	5 (Very severe)	92(13.1)	**5(4.4)**	24(11.9)	**63(16.4)**	
Heart beating quickly and out of control						0.205
	0 (No symptom)	274(39.1)	45(39.5)	64(31.8)	165(42.9)	
	1	120(17.1)	21(18.4)	36(17.9)	63(16.4)	
	2	98(14.0)	11(9.6)	37(18.4)	50(13.0)	
	3	96(13.7)	17(14.9)	34(16.9)	45(11.7)	
	4	73(10.4)	12(10.5)	22(10.9)	39(10.1)	
	5 (Very severe)	39(5.6)	8(7.0)	8(4.0)	23(6.0)	
Difficulty in sleeping						0.068
	0 (No symptom)	140(20.0)	21(18.4)	32(15.9)	87(22.6)	
	1	72(10.3)	11(9.6)	22(10.9)	39(10.1)	
	2	83(11.9)	14(12.3)	16(8.0)	53(13.8)	
	3	120(17.1)	20(17.5)	48(23.9)	52(13.5)	
	4	112(16.0)	23(20.2)	31(15.4)	58(15.1)	
	5 (Very severe)	173(24.7)	25(21.9)	52(25.9)	96(24.9)	
Aching in muscles and/or joints						0.828
	0 (No symptom)	107(15.3)	15(13.2)	27(13.4)	65(16.9)	
	1	99(14.1)	19(16.7)	28(13.9)	52(13.5)	
	2	88(12.6)	17(14.9)	29(14.4)	42(10.9)	
	3	108(15.4)	20(17.5)	30(14.9)	58(15.1)	
	4	113(16.1)	16(14.0)	30(14.9)	67(17.4)	
	5 (Very severe)	185(26.4)	27(23.7)	57(28.4)	101(26.2)	
Feeling a lack of energy						0.127
	0 (No symptom)	111(15.9)	12(10.5)	30(14.9)	69(17.9)	
	1	99(14.1)	15(13.2)	21(10.4)	63(16.4)	
	2	105(15.0)	17(14.9)	33(16.4)	55(14.3)	
	3	97(13.9)	17(14.9)	24(11.9)	56(14.5)	
	4	103(14.7)	19(16.7)	40(19.9)	44(11.4)	
	5 (Very severe)	185(26.4)	34(29.8)	53(26.4)	98(25.5)	
Perception of being useless						0.101
	0 (No symptom)	276(39.4)	36(31.6)	68(33.8)	172(44.7)	
	1	88(12.6)	14(12.3)	29(14.4)	45(11.7)	
	2	67(9.6)	11(9.6)	26(12.9)	30(7.8)	
	3	74(10.6)	19(16.7)	21(10.4)	34(8.8)	
	4	80(11.4)	14(12.3)	24(11.9)	42(10.9)	
	5 (Very severe)	115(16.4)	20(17.5)	33(16.4)	62(16.1)	
Feel anxious or nervous						0.357
	0 (No symptom)	97(13.9)	12(10.5)	23(11.4)	62(16.1)	
	1	120(17.1)	15(13.2)	34(16.9)	71(18.4)	
	2	106(15.1)	13(11.4)	37(18.4)	56(14.5)	
	3	114(16.3)	20(17.5)	32(15.9)	62(16.1)	
	4	108(15.4)	22 (19.3)	31(15.4)	55(14.3)	
	5 (Very severe)	155(22.1)	32 (28.1)	44(21.9)	79(20.5)	
Urine leaks						≤ **0.001**
	0 (No symptom)	423(60.4)	63(55.3)	107(53.2)	**253(65.7)**	
	1	90(12.9)	13(11.4)	**36(17.9)**	41(10.6)	
	2	58(8.3)	**17(14.9)**	22(10.9)	19(4.9)	
	3	42(6.0)	11(9.6)	12(6.0)	19(4.9)	
	4	42(6.0)	5(4.4)	15(7.5)	22(5.7)	
	5 (Very severe)	45(6.4)	5(4.4)	9(4.5)	31(8.1)	
Vaginal discomfort and/or dryness						≤ **0.001**
	0 (No symptom)	245(35.0)	**49(43.0)**	**91(45.3)**	105(27.3)	
	1	106(15.1)	22(19.3)	29(14.4)	55(14.3)	
	2	90(12.9)	14(12.3)	27(13.4)	49(12.7)	
	3	66(9.4)	11(9.6)	21(10.4)	34(8.8)	
	4	86(12.3)	11(9.6)	23(11.4)	52(13.5)	
	5 (Very severe)	107(15.3)	7(6.1)	10(5.0)	**90(23.4)**	
Skin dryness						≤ **0.001**
		82(11.7)	**28(24.6)**	23(11.4)	31(8.1)	
	1	103(14.7)	14(12.3)	29(14.4)	60(15.6)	
		118(16.9)	18(15.8)	40(19.9)	60(15.6)	
	3	118(16.9)	19(16.7)	41(20.4)	58(15.1)	
	4	122(17.4)	19(16.7)	42(20.9)	61(15.8)	
	5 (Very severe)	157(22.4)	16(14.0)	26(12.9)	**115(29.9)**	
CS-10 total score						0.399
	0 – 10	123(17.6)	26(22.8)	28(13.9)	69(17.9)	
	11 – 20	187(26.7)	24(21.1)	58(28.9)	105(27.3)	
	21 – 30	215(30.7)	35(30.7)	67(33.3)	113(29.4)	
	31 – 40	126(18.0)	22(19.3)	38(18.9)	66(17.1)	
	41 – 50	49(7.0)	7(6.1)	10(5.0)	32(8.3)	
Hot flashes and/or night sweats		2.00 [1.00 – 4.00]	1.00 [0.00 – 3.00]*	3.00 [1.00 – 4.00]**	2.00 [1.00 – 4.00]**	≤ **0.001**
Heart beating quickly and out of control		1.00 [0.00 – 3.00]	1.00 [0.00 – 3.00]	2.00 [0.00 – 3.00]	1.00 [0.00 – 3.00]	0.135
Difficulty in sleeping		3.00 [1.00 – 4.00]	3.00 [1.00 – 4.00]	3.00 [1.00 – 5.00]	3.00 [1.00 – 4.00]	0.310
Aching in muscles and/or joints		3.00 [1.00 – 5.00]	3.00 [1.00 – 4.00]	3.00 [1.00 – 5.00]	3.00 [1.00 – 5.00]	0.734
Feeling a lack of energy		3.00 [1.00 – 5.00]	3.00 [2.00 – 5.00]	3.00 [1.00 – 5.00]	3.00 [1.00 – 5.00]	0.055
Perception of being useless		1.00 [0.00 – 4.00]	2.00 [0.00 – 4.00]	2.00 [0.00 – 4.00]	1.00 [0.00 – 4.00]	**0.050**
Feel anxious or nervous		3.00 [1.00 – 4.00]	3.00 [2.00 – 5.00]*	3.00 [1.00 – 4.00]**	3.00 [1.00 – 4.00]**	**0.024**
Urine leaks		0.00 [0.00 – 2.00]	0.00 [0.00 – 2.00]	0.00 [0.00 – 2.00]	0.00 [0.00 – 1.00]	0.060
Vaginal discomfort and/or dryness		1.00 [0.00 – 4.00]	1.00 [0.00 – 3.00]*	1.00 [0.00 – 3.00]*	2.00 [0.00 – 4.00]**	≤ **0.001**
Skin dryness (appearance, texture, or tone)		3.00 [1.00 – 4.00]	2.00 [1.00 – 4.00]*	3.00 [1.00 – 4.00]*	3.00 [2.00 – 5.00]**	≤ **0.001**
CS-10 total score		22.00 [13.00 – 30.50]	22.00 [11.00 – 31.00]	23.00 [14.00 – 30.00]	22.00 [13.00 – 31.00]	0.844

Data presented as absolute (n) and relative (%) frequencies, or median [md] and interquartile range ([IQR], percentiles 25th and 75th). In all individual question, minimum and maximum, for all groups, were 0 and 5, respectively. Legend: p – statistical significance index.

*/** Different letters indicate differences among groups. *Chi-Squared test with adjusted residual analysis or Kruskal-Wallis test with Dunn post hoc, when applicable. Significance set at 5% for all analysis

The FSFI-6 data is presented in table 4. In summary, perimenopausal women showed ‘high’ or ‘very high’ levels of desire (20.9%), arousal (24.7% and 10.8%), lubrication (32.9% and 18.4%), satisfaction (15.4%), with "almost never or never" feeling pain (36.7%) during sexual intercourse. On contrary, the postmenopausal group had "very low" or "none" desire (31.7%), arousal (19.4%), lubrication (22.2%), orgasm (20.1% and 11.7%), satisfaction (29.1%), with "always" or "almost always" (23.5%) or mostly (17.3%) feeling pain (χ² ≤ 0.05 for all) during sexual intercourse. In fact, the postmenopausal group showed the worse median [IQR] for desire (2 [1 – 3]), arousal (3 [2 – 3]), lubrication (3 [2 – 4]), orgasm (3 [2 – 4]), satisfaction (3 [1 – 4]), pain (3 [2 – 4]), and the FSFI-6 total score (12 [7 – 18]), in relation to both, the pre- and the perimenopausal groups (KW ≤ 0.05 for all).

**Table 4:** 6-item Female Sexual Function Index (FSFI-6)

Variable		Total (n = 700)	Premenopausal (n = 114)	Perimenopausal (n = 201)	Postmenopausal (n = 385)	*p-value
**Desire**						≤ **0.001**
	Very low or none	177 (25.3)	18 (15.8)	37 (18.4)	**122 (31.7)**	
	Low	327 (33.9)	47 (41.2)	57 (28.4)	133 (34.5)	
	Moderate	186 (26.6)	31 (27.2)	56 (27.9)	99 (25.7)	
	High	80 (11.4)	14 (12.3)	**42 (20.9)**	24 (6.2)	
	Very high	20 (2.9)	4 (3.5)	9 (4.5)	7 (1.8)	
**Arousal ^#^**						≤ **0.001**
	Very low or none	71 (14.3)	12 (12.5)	12 (7.6)	**47 (19.4)**	
	Low	111 (22.4)	20 (20.8)	29 (18.4)	62 (25.6)	
	Moderate	187 (37.7)	35 (36.5)	61 (38.6)	91 (37.6)	
	High	95 (19.2)	23 (24.0)	**39 (24.7)**	33 (13.6)	
	Very high	32 (6.5)	6 (6.3)	**17 (10.8)**	9 (3.7)	
**Lubrication ^#^**						≤ **0.001**
	Almost never or never	71 (14.3)	8 (8.4)	9 (5.7)	**54 (22.2)**	
	Few times	104 (21.0)	23 (24.2)	27 (17.1)	54 (22.2)	
	Sometimes	125 (25.2)	25 (26.3)	41 (25.9)	59 (24.3)	
	Mostly	128 (25.8)	26 (27.4)	**52 (32.9)**	50 (20.6)	
	Always or almost always	68 (13.7)	13 (13.7)	**29 (18.4)**	26 (10.7)	
**Orgasm ^##^**						≤ **0.001**
	Almost never or never	49 (8.4)	7 (6.9)	6 (3.4)	**36 (11.7)**	
	Few times	89 (15.2)	8 (7.9)	19 (10.8)	**62 (20.1)**	
	Sometimes	148 (25.3)	30 (29.7)	46 (26.1)	72 (23.3)	
	Mostly	193 (32.9)	34 (33.7)	68 (38.6)	91 (29.4)	
	Always or almost always	107 (18.3)	22 (21.8)	37 (21.0)	48 (15.5)	
**Satisfaction**						**0.001**
	Very dissatisfied	159 (22.7)	18 (15.8)	29 (14.4)	**112 (29.1)**	
	Moderately dissatisfied	109 (15.6)	19 (16.7)	28 (13.9)	62 (16.1)	
	Neither satisfied nor dissatisfied	179 (25.6)	30 (26.3)	57 (28.4)	92 (23.9)	
	Moderately satisfied	175 (25.0)	34 (29.8)	56 (27.9)	85 (22.1)	
	Very satisfied	78 (11.1)	13 (11.4)	**31 (15.4)**	34 (8.8)	
**Pain ^#^**						≤ **0.001**
	Always or almost always	78 (15.7)	8 (8.8)	10 (6.7)	**60 (23.5)**	
	Mostly	63 (12.7)	9 (9.9)	10 (6.7)	**44 (17.3)**	
	Sometimes	120 (24.2)	26 (28.6)	38 (25.3)	56 (22.0)	
	Few times	110 (22.2)	27 (29.7)	37 (24.7)	56 (18.0)	
	Almost never or never	125 (25.2)	21 (23.1)	**55 (36.7)**	49 (19.2)	
**FSFI-6 total score**						≤ **0.001**
	≤ 21	564 (80.6)	85 (74.6)	138 (68.7)	**341 (88.6)**	
	≥ 22	136 (19.4)	29 (25.4)	**63 (31.3)**	44 (11.4)	
**Desire**		2.00 [1.00 – 3.00]	2.00 [2.00 – 3.00]*	3.00 [2.00 – 4.00]*	2.00 [1.00 – 3.00]**	≤ **0.001**
**Arousal ^#^**		3.00 [2.00 – 4.00]	3.00 [2.00 – 4.00]*	3.00 [2.00 – 4.00]*	3.00 [2.00 – 3.00]**	≤ **0.001**
**Lubrication ^#^**		3.00 [2.00 – 4.00]	3.00 [2.00 – 4.00]*	4.00 [3.00 – 4.00]*	3.00 [2.00 – 4.00]**	≤ **0.001**
**Orgasm ^##^**		4.00 [3.00 – 4.00]	4.00 [3.00 – 4.00]*	4.00 [3.00 – 4.00]*	3.00 [2.00 – 4.00]**	≤ **0.001**
**Satisfaction**		3.00 [2.00 – 4.00]	3.00 [2.00 – 4.00]*	3.00 [2.00 – 4.00]*	3.00 [1.00 – 4.00]**	≤ **0.001**
**Pain ^#^**		3.00 [2.00 – 5.00]	4.00 [3.00 – 4.00]*	4.00 [3.00 – 5.00]*	3.00 [2.00 – 4.00]**	≤ **0.001**
**FSFI-6 total score**		15.00 [8.00 – 20.00]	17.50 [12.00 – 22.00]*	18.00 [11.5 – 23.00]*	12.00 [7.00 – 18.00]**	≤ **0.001**

Data presented as absolute (n) and relative (%) frequencies, or median [md] and interquartile range ([IQR], percentiles 25th and 75th). In all individual question, minimum and maximum, for all groups, were 1 and 5, respectively. Legend: p – statistical significance index.

*/** Indicate differences among groups. *Chi-Squared test with adjusted residual analysis or Kruskal-Wallis test with Dunn post hoc, when applicable. Significance set at 5% for all analysis.

# Total N = 496 (70.9%).

## Total N = 586 (83,7%).

The WHO VAW questionnaire is displayed in table 5. Although the prevalence rates were not significantly different among the assessed groups, we would like to emphasize that 36.9% of participants reported having already suffered some type of violence, in many cases being committed by someone they already knew (family member, neighbor, friend) (74.1%). The proportions of physical (21.3%), sexual (34.9%), and psychological (38.8%) violences were similar among groups. The premenopausal group was associated with higher prevalence of being hit with his/her fist or with some object (14.0%), while the postmenopausal group was not associated with such form of violence (94.5%) (χ² = 0.009). In addition, 22.7% of the perimenopausal group reported that, in the last 12 months, someone forced her to have sex against her will (χ² = 0.037).

**Table 5:** Experienced violence and Violence against women questionnaire (WHO VAW)

Variable		Total (n = 700)	Premenopausal (n = 114)	Perimenopausal (n = 201)	Postmenopausal (n = 385)	*p-value
Have ever suffered any type of violence?						0.779
	No	442 (63.1)	71 (62.3)	131 (65.2)	240 (62.3)	
	Yes	258 (36.9)	43 (37.7)	70 (34.8)	145 (37.7)	
Who committed the violence						0.381
	Known person (family, neighbor, friend)	152 (74.1)	29 (78.4)	37 (67.3)	86 (76.1)	
	Unknown person	53 (25.9)	8 (21.6)	18 (32.7)	27 (23.9)	
Type of violence						0.376
	Physical	77 (21.3)	14 (23.3)	17 (16.2)	46 (23.5)	
	Sexual	126 (34.9)	23 (38.3)	35 (33.3)	68 (34.7)	
	Psychological	140 (38.8)	21 (35.0)	44 (41.9)	75 (38.3)	
	Other	18 (5.0)	2 (3.3)	9 (8.6)	7 (3.6)	
Previous family violence						0.631
	No	155 (42.9)	29 (48.3)	45 (42.9)	81 (41.3)	
	Yes	206 (57.1)	31 (51.7)	60 (57.1)	115 (58.7)	
Psychological violence						
Insulted me						0.922
	No	388 (55.4)	62 (54.4)	110 (54.7)	216 (56.1)	
	Yes	312 (44.6)	52 (45.6)	91 (45.3)	169 (43.9)	
In the last 12 months						0.575
	No	209 (67.0)	36 (69.2)	57 (62.6)	116 (68.6)	
	Yes	103 (33.0)	16 (30.8)	34 (37.4)	53 (31.4)	
In the last 12 months						0.307
	Once	17 (16.5)	5 (31.3)	5 (14.7)	7 (13.2)	
	Few times	56 (54.4)	8 (50.0)	21 (61.8)	27 (50.9)	
	Many times	30 (29.1)	3 (18.8)	8 (23.5)	19 (35.8)	
Before the last 12 months						0.299
	Once	15 (14.6)	2 (12.5)	8 (23.5)	5 (9.4)	
	Few times	56 (54.4)	9 (56.3)	19 (55.9)	28 (52.8)	
	Many times	32 (31.1)	5 (31.3)	7 (20.6)	20 (37.7)	
Belittled or humiliated me						0.862
	No	508 (72.6)	83 (72.8)	143 (71.1)	282 (73.2)	
	Yes	192 (27.4)	31 (27.2)	58 (28.9)	103 (26.8)	
In the last 12 months						0.857
	No	128 (66.7)	21 (67.7)	37 (63.8)	70 (68.0)	
	Yes	64 (33.3)	10 (32.3)	21 (36.2)	33 (32.0)	
In the last 12 months						0.356
	Once	11 (17.2)	1 (10.0)	6 (28.6)	4 (12.1)	
	Few times	39 (60.9)	8 (80.0)	10 (47.6)	21 (63.6)	
	Many times	14 (21.9)	1 (10.0)	5 (23.8)	8 (24.2)	
Before the last 12 months						0.643
	Once	9 (14.1)	1 (10.0)	5 (23.8)	3 (9.1)	
	Few times	35 (54.7)	6 (60.0)	10 (47.6)	19 (57.6)	
	Many times	20 (31.3)	3 (30.0)	6 (28.6)	11 (33.3)	
Tried to scare or intimidate me						0.905
	No	515 (73.6)	82 (71.9)	148 (73.6)	285 (74.0)	
	Yes	185 (26.4)	32 (28.1)	53 (26.4)	100 (26.0)	
In the last 12 months						0.843
	No	138 (74.6)	24 (75.0)	38 (71.7)	76 (76.0)	
	Yes	47 (25.4)	8 (25.0)	15 (28.3)	24 (24.0)	
In the last 12 months						0.668
	Once	6 (12.8)	1 (12.5)	1 (6.7)	4 (16.7)	
	Few times	27 (57.4)	6 (75.0)	9 (60.0)	12 (50.0)	
	Many times	14 (29.8)	1 (12.5)	5 (33.3)	8 (33.3)	
Before the last 12 months						0.628
	Once	6 (12.8)	1 (12.5)	1 (6.7)	4 (16.7)	
	Few times	20 (42.6)	5 (62.5)	6 (40.0)	9 (37.5)	
	Many times	21 (44.7)	2 (25.0)	8 (53.3)	11 (45.8)	
Threatened to hurt me or someone I care						0.269
	No	596 (85.1)	96 (84.2)	165 (82.1)	335 (87.0)	
	Yes	104 (14.9)	18 (15.8)	36 (17.9)	50 (13.0)	
In the last 12 months						0.289
	No	79 (76.0)	16 (88.9)	25 (69.4)	38 (76.0)	
	Yes	25 (24.0)	2 (11.1)	11 (30.6)	12 (24.0)	
In the last 12 months						0.596
	Once	4 (16.0)	0 (0.0)	3 (27.3)	1 (8.3)	
	Few times	16 (64.0)	2 (100.0)	6 (54.5)	8 (66.7)	
	Many times	5 (20.0)	0 (0.0)	2 (18.2)	3 (25.0)	
Before the last 12 months						0.494
	Once	5 (20.0)	0 (0.0)	2 (18.2)	3 (25.0)	
	Few times	12 (48.0)	2 (100.0)	6 (54.5)	4 (33.3)	
	Many times	8 (32.0)	0 (0.0)	3 (27.3)	5 (41.7)	
Physical violence						
Slapped me or threw something at me						0.105
	No	599 (85.6)	91 (79.8)	178 (88.6)	330 (85.7)	
	Yes	101 (14.4)	23 (20.2)	23 (11.4)	55 (14.3)	
In the last 12 months						0.438
	No	89 (88.1)	22 (95.7)	20 (87.0)	47 (85.5)	
	Yes	12 (11.9)	1 (4.3)	3 (13.0)	8 (14.5)	
In the last 12 months						0.292
	Once	5 (41.7)	1 (100.0)	0 (0.0)	4 (50.0)	
	Few times	6 (50.0)	0 (0.0)	3 (100.0)	3 (37.5)	
	Many times	1 (8.3)	0 (0.0)	0 (0.0)	1 (12.5)	
Before the last 12 months						0.462
	Once	5 (41.7)	1 (100.0)	1 (33.3)	3 (37.5)	
	Few times	7 (58.3)	0 (0.0)	2 (66.7)	5 (62.5)	
	Many times	0 (0.0)	0 (0.0)	0 (0.0)	0 (0.0)	
Pushed or shoved me						0.382
	No	577 (82.4)	89 (78.1)	169 (84.1)	319 (82.9)	
	Yes	123 (17.6)	25 (21.9)	32 (15.9)	66 (17.1)	
In the last 12 months						0.496
	No	104 (84.6)	23 (92.0)	26 (81.3)	55 (83.3)	
	Yes	19 (15.4)	2 (8.0)	6 (18.8)	11 (16.7)	
In the last 12 months						0.671
	Once	10 (52.6)	1 (50.0)	3 (50.0)	6 (54.5)	
	Few times	8 (42.1)	1 (50.0)	2 (33.3)	5 (45.5)	
	Many times	1 (5.3)	0 (0.0)	1 (16.7)	0 (0.0)	
Before the last 12 months						0.286
	Once	7 (36.8)	1 (50.0)	2 (33.3)	4 (36.4)	
	Few times	10 (52.6)	0 (0.0)	4 (66.7)	6 (54.5)	
	Many times	2 (10.5)	1 (50.0)	0 (0.0)	1 (9.1)	
Hit me with his/her fist or some object						**0.009**
	No	647 (92.4)	98 (86.0)	185 (92.0)	**364 (94.5)**	
	Yes	53 (7.6)	**16 (14.0)**	16 (8.0)	21 (5.5)	
In the last 12 months						0.308
	No	52 (98.1)	15 (93.8)	16 (100.0)	21 (100.0)	
	Yes	1 (1.9)	1 (6.3)	0 (0.0)	0 (0.0)	
In the last 12 months						1.000
	Once	0 (0.0)	0 (0.0)	0 (0.0)	0 (0.0)	
	Few times	1 (100.0)	1 (100.0)	0 (0.0)	0 (0.0)	
	Many times	0 (0.0)	0 (0.0)	0 (0.0)	0 (0.0)	
Before the last 12 months						1.000
	Once	0 (0.0)	0 (0.0)	0 (0.0)	0 (0.0)	
	Few times	1 (100.0)	1 (100.0)	0 (0.0)	0 (0.0)	
	Many times	0 (0.0)	0 (0.0)	0 (0.0)	0 (0.0)	
Kicked or dragged me or beat me up						0.194
	No	653 (93.3)	102 (89.5)	190 (94.5)	361 (93.8)	
	Yes	47 (6.7)	12 (10.5)	11 (5.5)	24 (6.2)	
In the last 12 months						0.167
	No	44 (93.6)	12 (100.0)	9 (81.8)	23 (95.8)	
	Yes	3 (6.4)	0 (0.0)	2 (18.2)	1 (4.2)	
In the last 12 months						1.000
	Once	3 (100.0)	0 (0.0)	2 (100.0)	1 (100.0)	
	Few times	0 (0.0)	0 (0.0)	0 (0.0)	0 (0.0)	
	Many times	0 (0.0)	0 (0.0)	0 (0.0)	0 (0.0)	
Before the last 12 months						1.000
	Once	3 (100.0)	0 (0.0)	2 (100.0)	1 (100.0)	
	Few times	0 (0.0)	0 (0.0)	0 (0.0)	0 (0.0)	
	Many times	0 (0.0)	0 (0.0)	0 (0.0)	0 (0.0)	
Choked me or burnt me on purpose						0.619
	No	679 (97.0)	109 (95.6)	196 (97.5)	374 (97.1)	
	Yes	21 (3.0)	5 (4.4)	5 (2.5)	11 (2.9)	
In the last 12 months						1.000
	No	21 (100.0)	5 (100.0)	5 (100.0)	11 (100.0)	
	Yes	0 (0.0)	0 (0.0)	0 (0.0)	0 (0.0)	
In the last 12 months						1.000
	Once	0 (0.0)	0 (0.0)	0 (0.0)	0 (0.0)	
	Few times	0 (0.0)	0 (0.0)	0 (0.0)	0 (0.0)	
	Many times	0 (0.0)	0 (0.0)	0 (0.0)	0 (0.0)	
Before the last 12 months						1.000
	Once	0 (0.0)	0 (0.0)	0 (0.0)	0 (0.0)	
	Few times	0 (0.0)	0 (0.0)	0 (0.0)	0 (0.0)	
	Many times	0 (0.0)	0 (0.0)	0 (0.0)	0 (0.0)	
Hurt me with a knife, gun or other weapon						0.576
	No	669 (95.6)	111 (97.4)	192 (95.5)	366 (95.1)	
	Yes	31 (4.4)	3 (2.6)	9 (4.5)	19 (4.9)	
In the last 12 months						0.750
	No	29 (93.5)	3 (100.0)	8 (88.9)	18 (94.7)	
	Yes	2 (6.5)	0 (0.0)	1 (11.1)	1 (5.3)	
In the last 12 months						0.157
	Once	1 (50.0)	0 (0.0)	0 (0.0)	1 (100.0)	
	Few times	0 (0.0)	0 (0.0)	0 (0.0)	0 (0.0)	
	Many times	1 (50.0)	0 (0.0)	1 (100.0)	0 (0.0)	
Before the last 12 months						0.157
	Once	1 (50.0)	0 (0.0)	0 (0.0)	1 (100.0)	
	Few times	1 (50.0)	0 (0.0)	1 (100.0)	0 (0.0)	
	Many times	0 (0.0)	0 (0.0)	0 (0.0)	0 (0.0)	
Sexual violence						
Demanded to have sexual intercourse						0.524
	No	608 (86.9)	97 (85.1)	179 (89.1)	332 (86.2)	
	Yes	92 (13.1)	17 (14.9)	22 (10.9)	53 (13.8)	
In the last 12 months						0.469
	No	86 (93.5)	17 (100.0)	20 (90.9)	49 (92.5)	
	Yes	6 (6.5)	0 (0.0)	2 (9.1)	4 (7.5)	
In the last 12 months						0.472
	Once	1 (16.7)	0 (0.0)	0 (0.0)	1 (25.0)	
	Few times	4 (66.7)	0 (0.0)	2 (100.0)	2 (50.0)	
	Many times	1 (16.7)	0 (0.0)	0 (0.0)	1 (25.0)	
Before the last 12 months						0.221
	Once	0 (0.0)	0 (0.0)	0 (0.0)	0 (0.0)	
	Few times	4 (66.7)	0 (0.0)	2 (100.0)	2 (50.0)	
	Many times	2 (33.3)	0 (0.0)	0 (0.0)	2 (50.0)	
Forced me to have sex against my will						0.830
	No	616 (88.0)	99 (86.8)	179 (89.1)	338 (87.8)	
	Yes	84 (12.0)	15 (13.2)	22 (10.9)	47 (12.2)	
In the last 12 months						**0.037**
	No	76 (90.5)	15 (100.0)	17 (77.3)	44 (93.6)	
	Yes	8 (9.5)	0 (0.0)	**5 (22.7)**	3 (6.4)	
In the last 12 months						0.850
	Once	0 (0.0)	0 (0.0)	0 (0.0)	0 (0.0)	
	Few times	5 (62.5)	0 (0.0)	3 (60.0)	2 (66.7)	
	Many times	3 (37.5)	0 (0.0)	2 (40.0)	1 (33.3)	
Before the last 12 months						0.465
	Once	0 (0.0)	0 (0.0)	0 (0.0)	0 (0.0)	
	Few times	4 (50.0)	0 (0.0)	3 (60.0)	1 (33.3)	
	Many times	4 (50.0)	0 (0.0)	2 (40.0)	2 (66.7)	
Forced me to do degrading sexual acts						0.704
	No	653 (93.3)	106 (93.0)	190 (94.5)	357 (92.7)	
	Yes	47 (6.7)	8 (7.0)	11 (5.5)	28 (7.3)	
In the last 12 months						0.492
	No	45 (95.7)	8 (100.0)	11 (100.0)	26 (92.9)	
	Yes	2 (4.3)	0 (0.0)	0 (0.0)	2 (7.1)	
In the last 12 months						1.000
	Once	0 (0.0)	0 (0.0)	0 (0.0)	0 (0.0)	
	Few times	2 (100.0)	0 (0.0)	0 (0.0)	2 (100.0)	
	Many times	0 (0.0)	0 (0.0)	0 (0.0)	0 (0.0)	
Before the last 12 months						1.000
	Once	0 (0.0)	0 (0.0)	0 (0.0)	0 (0.0)	
	Few times	2 (100.0)	0 (0.0)	0 (0.0)	2 (100.0)	
	Many times	0 (0.0)	0 (0.0)	0 (0.0)	0 (0.0)	
VAW total score		1.00 [0.00 – 3.00]	1.00 [0.00 – 3.00]	1.00 [0.00 – 3.00]	1.00 [0.00 – 3.00]	0.865
Psychological VAW total score		0.00 [0.00 – 2.00]	1.00 [0.00 – 2.00]	1.00 [0.00 – 2.00]	0.00 [0.00 – 2.00]	0.834
Physical VAW total score		0.00 [0.00 – 0.00]	0.00 [0.00 – 1.00]	0.00 [0.00 – 0.00]	0.00 [0.00 – 0.00]	0.414
Sexual VAW total score		0.00 [0.00 – 0.00]	0.00 [0.00 – 0.00]	0.00 [0.00 – 0.00]	0.00 [0.00 – 0.00]	0.607

Data presented as absolute (n) and relative (%) frequencies. p – statistical significance index. Numbers highlighted in bold indicate association between categories.

* Chi-Squared test with adjusted residual analysis or Kruskal-Wallis test with Dunn post hoc, when applicable. Significance set at 5% for all analysis

Table 6 provides a detailed overview of lifestyle and habits, highlighting significant differences between individuals exposed to domestic violence and those not exposed. Statistically significant findings include a higher prevalence of ex-smokers among violence-exposed individuals (30.2%) compared to non-smokers among those not exposed (73.8%) (χ² ≤ 0.001). Additionally, a significantly higher proportion of violence-exposed individuals reported using illicit drugs in the past year (8.9%) compared to the high percentage of non-users among those not exposed (97.3%) (χ² ≤ 0.001). Furthermore, the use of unprescribed tranquilizers was significantly more common among violence-exposed individuals (3.1%) compared to those not exposed (0.5%) (χ² = 0.004), and marijuana/hash/skank use was notably higher among the violence-exposed group (7.4%) (χ² ≤ 0.001).

**Table 6:** Lifestyle and habits characterization

Variable		Total (n = 700)	NV (n = 442)	V (n = 258)	*p-value
Smoking					≤ **0.001**
	No	476 (68.0)	326 (73.8)	150 (58.1)	
	Yes	68 (9.7)	38 (8.6)	30 (11.6)	
	Ex-smoker	156 (22.3)	78 (17.6)	**78 (30.2)**	
Cigarettes per day					0.396
	Less than 5	16 (23.5)	9 (23.7)	7 (23.3)	
	5 to 10	20 (29.4)	12 (31.6)	8 (26.7)	
	11 to 20	26 (38.2)	12 (31.6)	14 (46.7)	
	More than 20	6 (8.8)	5 (13.2)	1 (3.3)	
Illicit drug (last year)					≤ **0.001**
	No	665 (95.0)	430 (97.3)	235 (91.1)	
	Yes	35 (5.0)	12 (2.7)	**23 (8.9)**	
Illicit drug (last year)					
	Cocaine	4 (0.6)	2 (0.5)	2 (0.8)	0.585
	Aspirated Ritalin	1 (0.1)	0 (0.0)	1 (0.4)	0.190
	Unprescribed tranquilizers	10 (1.4)	2 (0.5)	8 (3.1)	0.004
	Marijuana, Hash or Skank	28 (4.0)	9 (2.0)	**19 (7.4)**	≤ **0.001**
Alcohol intake					0.205
	Never drink	205 (29.3)	139 (31.4)	66 (25.6)	
	1 to 2 times a month	254 (36.3)	148 (33.5)	106 (41.1)	
	1 to 2 times a week	223 (31.9)	142 (32.1)	81 (31.4)	
	Once a day	16 (2.3)	11 (2.5)	5 (1.9)	
	More than once a day	2 (0.3)	2 (0.5)	0 (0.0)	
Coffee intake – n (%)					0.617
	No	76 (10.9)	46 (10.4)	30 (11.6)	
	Yes	624 (89.1)	396 (89.6)	228 (88.4)	
Coffee intake (daily) – n (%)					0.083
	Occasionally	55 (8.8)	36 (9.1)	19 (8.3)	
	1 to 2 coffees	330 (53.0)	212 (53.7)	118 (51.8)	
	3 to 4 coffees	196 (31.5)	114 (28.9)	82 (36.0)	
	More than 5 coffees	42 (6.7)	33 (8.4)	9 (3.9)	

Data presented as absolute (n) and relative (%) frequencies. NV – group of women who reported not having suffered any violence. V – group of women who reported having suffered any violence. p – statistical significance index. Numbers highlighted in bold indicate association between categories.

* Chi-Squared test with adjusted residual analysis. Significance set at 5% for all analysis

Table 7 illustrates the results of the CS-10 assessing various symptoms among individuals exposed to domestic violence compared to those not exposed. Notably, there are statistically significant differences in the perception of being useless and feeling anxious or nervous, with higher proportions and median scores observed in the violence-exposed group (χ² or KW ≤ 0.001 for both). Specifically, violence-exposed individuals reported a greater perception aching in muscles and/or joints (median [IQR] score of 3 [1 – 5] vs. 3 [1 – 4]), being useless (median [IQR] score of 2 [0 – 4] vs. 1 [0 – 3]) and higher levels of anxiety or nervousness (median [IQR] score of 3 [2 – 5] vs. 2 [1 – 4]). Additionally, the total CS-10 score was significantly higher for the violence-exposed group (median [IQR] score of 24 [15 – 32]) compared to those not exposed (median [IQR] score of 22 [12 – 30]) (KW = 0.009).

**Table 7:** 10-item Cervantes Scale (CS-10)

Variable		Total (n = 700)	NV (n = 442)	V (n = 258)	*p-value
Hot flashes and/or night sweats					0.941
	0 (No symptom)	171 (24.4)	110 (24.9)	61 (23.6)	
	1	108 (15.4)	64 (14.5)	44 (17.1)	
	2	95 (13.6)	59 (13.3)	36 (14.0)	
	3	109 (15.6)	72 (16.3)	37 (14.3)	
	4	125 (17.9)	79 (17.9)	46 (17.8)	
	5 (Very severe)	92 (13.1)	58 (13.1)	34 (13.2)	
Heart beating quickly and out of control					0.078
	0 (No symptom)	274 (39.1)	185 (41.9)	89 (34.5)	
	1	120 (17.1)	63 (14.3)	57 (22.1)	
	2	98 (14.0)	67 (15.2)	31 (12.0)	
	3	96 (13.7)	57 (12.9)	39 (15.1)	
	4	73 (10.4)	45 (10.2)	28 (10.9)	
	5 (Very severe)	39 (5.6)	25 (5.7)	14 (5.4)	
Difficulty in sleeping					0.346
	0 (No symptom)	140 (20.0)	86 (19.5)	54 (20.9)	
	1	72 (10.3)	49 (11.1)	23 (8.9)	
	2	83 (11.9)	60 (13.6)	23 (8.9)	
	3	120 (17.1)	70 (15.8)	50 (19.4)	
	4	112 (16.0)	72 (16.3)	40 (15.5)	
	5 (Very severe)	173 (24.7)	105 (23.8)	68 (26.4)	
Aching in muscles and/or joints					0.128
	0 (No symptom)	107 (15.3)	72 (16.3)	35 (13.6)	
	1	99 (14.1)	61 (13.8)	38 (14.7)	
	2	88 (12.6)	64 (14.5)	24 (9.3)	
	3	108 (15.4)	71 (16.1)	37 (14.3)	
	4	113 (16.1)	70 (15.8)	43 (16.7)	
	5 (Very severe)	185 (26.4)	104 (23.5)	81 (31.4)	
Feeling a lack of energy					0.354
	0 (No symptom)	111 (15.9)	73 (16.5)	38 (14.7)	
	1	99 (14.1)	65 (14.7)	34 (13.2)	
	2	105 (15.0)	65 (14.7)	40 (15.5)	
	3	97 (13.9)	67 (15.2)	30 (11.6)	
	4	103 (14.7)	67 (15.2)	36 (14.0)	
	5 (Very severe)	185 (26.4)	105 (23.8)	80 (31.0)	
Perception of being useless					≤ **0.001**
	0 (No symptom)	276 (39.4)	196 (44.3)	80 (31.0)	
	1	88 (12.6)	62 (14.0)	26 (10.1)	
	2	67 (9.6)	39 (8.8)	38 (10.9)	
	3	74 (10.6)	38 (8.6)	36 (14.0)	
	4	80 (11.4)	49 (11.1)	31 (12.0)	
	5 (Very severe)	115 (16.4)	58 (13.1)	**57 (22.1)**	
Feel anxious or nervous					≤ **0.001**
	0 (No symptom)	97 (13.9)	72 (16.3)	25 (9.7)	
	1	120 (17.1)	86 (19.5)	34 (13.2)	
	2	106 (15.1)	64 (14.5)	42 (16.3)	
	3	114 (16.3)	76 (17.2)	38 (14.7)	
	4	108 (15.4)	65 (14.7)	43 (16.7)	
	5 (Very severe)	155 (22.1)	79 (17.9)	**76 (29.5)**	
Urine leaks					0.400
	0 (No symptom)	423 (60.4)	274 (62.0)	149 (57.8)	
	1	90 (12.9)	56 (12.7)	34 (13.2)	
	2	58 (8.3)	39 (8.8)	19 (7.4)	
	3	42 (6.0)	25 (5.7)	17 (6.6)	
	4	42 (6.0)	26 (5.9)	16 (6.2)	
	5 (Very severe)	45 (6.4)	22 (5.0)	23 (8.9)	
Vaginal discomfort and/or dryness					0.411
	0 (No symptom)	245 (35.0)	150 (33.9)	95 (36.8)	
	1	106 (15.1)	74 (16.7)	32 (12.4)	
	2	90 (12.9)	56 (12.7)	34 (13.2)	
	3	66 (9.4)	36 (8.1)	30 (11.6)	
	4	86 (12.3)	55 (12.4)	31 (12.0)	
	5 (Very severe)	107 (15.3)	71 (16.1)	36 (14.0)	
Skin dryness (appearance, texture, or tone)					0.380
	0 (No symptom)	82 (11.7)	57 (12.9)	25 (9.7)	
	1	103 (14.7)	67 (15.2)	36 (14.0)	
	2	118 (16.9)	77 (17.4)	41 (15.9)	
	3	118 (16.9)	68 (15.4)	50 (19.4)	
	4	122 (17.4)	81 (18.3)	41 (15.9)	
	5 (Very severe)	157 (22.4)	92 (20.8)	65 (25.2)	
CS-10 total score					0.159
	0 – 10	123 (17.6)	89 (20.1)	34 (13.2)	
	11 – 20	187 (26.7)	119 (26.9)	68 (26.4)	
	21 – 30	215 (30.7)	132 (29.9)	83 (32.2)	
	31 – 40	126 (18.0)	73 (16.5)	53 (20.5)	
	41 – 50	49 (7.0)	29 (6.6)	20 (7.8)	
Hot flashes and/or night sweats		2.00 [1.00 – 4.00]	2.00 [1.00 – 4.00]	2.00 [1.00 – 4.00]	0.922
Heart beating quickly and out of control		1.00 [0.00 – 3.00]	1.00 [0.00 – 3.00]	100 [0.00 – 3.00]	0.271
Difficulty in sleeping		3.00 [1.00 – 4.00]	3.00 [1.00 – 4.00]	3.00 [1.00 – 5.00]	0.511
Aching in muscles and/or joints		3.00 [1.00 – 5.00]	3.00 [1.00 – 4.00]	3.00 [1.00 – 5.00]	**0.036**
Feeling a lack of energy		3.00 [1.00 – 5.00]	3.00 [1.00 – 4.00]	3.00 [1.00 – 5.00]	0.119
Perception of being useless		1.00 [0.00 – 4.00]	1.00 [0.00 – 3.00]	2.00 [0.00 – 4.00]	≤ **0.001**
Feel anxious or nervous		3.00 [1.00 – 4.00]	2.00 [1.00 – 4.00]	3.00 [2.00 – 5.00]	≤ **0.001**
Urine leaks		0.00 [0.00 – 2.00]	0.00 [0.00 – 2.00]	0.00 [0.00 – 2.00]	0.157
Vaginal discomfort and/or dryness		1.00 [0.00 – 4.00]	1.00 [0.00 – 4.00]	2.00 [0.00 – 4.00]	0.656
Skin dryness (appearance, texture, or tone)		3.00 [1.00 – 4.00]	3.00 [1.00 – 4.00]	3.00 [2.00 – 5.00]	0.135
CS-10 total score		22.00 [13.00 – 30.50]	22.00 [12.00 – 30.00]	24.00 [15.00 – 32.00]	**0.009**

Data presented as absolute (n) and relative (%) frequencies, or median [md] and interquartile range ([IQR], percentiles 25th and 75th). In all individual question, minimum and maximum, for all groups, were 0 and 5, respectively. Legend: NV – group of women who reported not having suffered any violence. V – group of women who reported having suffered any violence. p – statistical significance index. Numbers highlighted in bold indicate association between categories. / Indicate differences among groups.

* Chi-Squared test with adjusted residual analysis or Mann-Whitney test, when applicable. Significance set at 5% for all analysis

Table 8 presents data on the FSFI-6, comparing sexual function between women exposed to domestic violence and those not exposed. The analysis found no significant differences between the two groups across the subscales of desire, arousal, lubrication, orgasm, satisfaction, or pain (χ² or MW > 0.05). However, a statistically significant difference was identified in the total FSFI-6 score (KW = 0.041), with women exposed to violence showing a lower overall score (median [IQR] of 13 [7–20]) compared to those not exposed (median [IQR] of 16 [9–20]).

**Table 8:** 6-item Female Sexual Function Index (FSFI-6)

Variable		Total (n = 700)	NV (n = 442)	V (n = 258)	*p-value
Desire					0.853
	Very low or none	177 (25.3)	110 (24.9)	67 (26.0)	
	Low	327 (33.9)	155 (35.1)	82 (31.8)	
	Moderate	186 (26.6)	118 (26.7)	68 (26.4)	
	High	80 (11.4)	47 (10.6)	33 (12.8)	
	Very high	20 (2.9)	12 (2.7)	8 (3.1)	
Arousal ^#^					0.175
	Very low or none	71 (14.3)	43 (13.0)	28 (17.1)	
	Low	111 (22.4)	79 (23.8)	32 (19.5)	
	Moderate	187 (37.7)	133 (40.1)	54 (32.9)	
	High	95 (19.2)	59 (17.8)	36 (22.0)	
	Very high	32 (6.5)	18 (5.4)	14 (8.5)	
Lubrication ^#^					0.705
	Almost never or never	71 (14.3)	45 (13.6)	26 (15.8)	
	Few times	104 (21.0)	75 (22.7)	29 (17.6)	
	Sometimes	125 (25.2)	80 (24.2)	45 (27.3)	
	Mostly	128 (25.8)	85 (25.7)	43 (26.1)	
	Always or almost always	68 (13.7)	46 (13.9)	22 (13.3)	
Orgasm ^##^					0.211
	Almost never or never	49 (8.4)	28 (7.4)	21 (10.2)	
	Few times	89 (15.2)	56 (14.7)	33 (16.0)	
	Sometimes	148 (25.3)	107 (28.2)	41 (19.9)	
	Mostly	193 (32.9)	124 (32.6)	69 (33.5)	
	Always or almost always	107 (18.3)	65 (17.1)	42 (20.4)	
Satisfaction					0.666
	Very dissatisfied	159 (22.7)	97 (21.9)	62 (24.0)	
	Moderately dissatisfied	109 (15.6)	65 (14.7)	44 (17.1)	
	Neither satisfied nor dissatisfied	179 (25.6)	114 (25.8)	65 (25.2)	
	Moderately satisfied	175 (25.0)	118 (26.7)	57 (22.1)	
	Very satisfied	78 (11.1)	48 (10.9)	30 (11.6)	
Pain ^#^					0.822
	Always or almost always	78 (15.7)	52 (16.0)	26 (15.2)	
	Mostly	63 (12.7)	37 (11.4)	26 (15.2)	
	Sometimes	120 (24.2)	79 (24.3)	41 (24.0)	
	Few times	110 (22.2)	74 (22.8)	36 (21.1)	
	Almost never or never	125 (25.2)	83 (25.5)	42 (24.6)	
FSFI-6 total score					0.941
	≤ 21	564 (80.6)	357 (80.8)	207 (80.2)	
	≥ 22	136 (19.4)	85 (19.2)	51 (19.8)	
Desire		2.00 [1.00 – 3.00]	2.00 [2.00 – 3.00]	2.00 [1.00 – 3.00]	0.694
Arousal ^#^		3.00 [2.00 – 4.00]	3.00 [2.00 – 3.00]	3.00 [2.00 – 4.00]	0.506
Lubrication ^#^		3.00 [2.00 – 4.00]	3.00 [2.00 – 4.00]	3.00 [2.00 – 4.00]	0.960
Orgasm ^##^		4.00 [3.00 – 4.00]	3.00 [3.00 – 4.00]	4.00 [2.00 – 4.00]	0.715
Satisfaction		3.00 [2.00 – 4.00]	3.00 [2.00 – 4.00]	3.00 [2.00 – 4.00]	0.332
Pain ^#^		3.00 [2.00 – 5.00]	3.00 [2.00 – 5.00]	3.00 [2.00 – 4.00]	0.625
FSFI-6 total score		15.00 [8.00 – 20.00]	16.00 [9.00 – 20.00]	13.00 [7.00 – 20.00]	**0.041**

Data presented as absolute (n) and relative (%) frequencies, or median [md] and interquartile range ([IQR], percentiles 25th and 75th). In all individual question, minimum and maximum, for all groups, were 1 and 5, respectively. NV – group of women who reported not having suffered any violence. V – group of women who reported having suffered any violence. p – statistical significance index. Numbers highlighted in bold indicate association between categories. / Indicate differences among groups.

* Chi-Squared test with adjusted residual analysis or Mann-Whitney test, when applicable. Significance set at 5% for all analysis.

# Total N = 496 (70.9%).

## Total N = 586 (83.7%)

Table 9 presents data from the WHO VAW questionnaire, highlighting the prevalence and types of violence experienced by the participants. Key findings indicate that most perpetrators were known individuals (74.1%), with reports of physical (21.3%), sexual (34.9%), and psychological (38.8%) violence. Psychological violence, such as insults (44.6%), belittlement/humiliation (27.4%), scare or intimidate (57.0%) and threats to harm the individual or someone they care about (14.9%), showed significant differences between groups, with higher frequencies reported by those exposed to violence. Physical violence, including slapping (14.4%), pushing (17.6%), being hit (7.6%), kicked or dragged (6.7%), choked or burnt (3.0%), and being hurt with a knife, gun, or other weapon (4.4%), was also notably prevalent among the exposed group, indicating strong associations. Regarding sexual violence, significant differences between groups were found for experiences such as being demanded to have sexual intercourse (13.1%), being forced to have sex against their will (12.0%), and being forced to engage in degrading sexual acts (6.7%). Additionally, all scores (WHO VAW total score, psychological total score, physical total score, and sexual total score) were higher in the group exposed to domestic/familiar violence.

**Table 9:** Experienced violence and Violence against women (VAW) questionnaire

Variable		Total (n = 700)	NV (n = 442)	V (n = 258)	*p-value
Who committed the violence					0.358
	Known person (family, neighbor, friend)	152 (74.1)	37 (68.5)	115 (76.2)	
	Unknown person	53 (25.9)	17 (31.5)	36 (23.8)	
Type of violence					≤ **0.001**
	Physical	77 (21.3)	23 (18.1)	54 (23.1)	
	Sexual	126 (34.9)	18 (14.2)	108 (46.2)	
	Psychological	140 (38.8)	76 (59.8)	64 (27.4)	
	Other	18 (5.0)	10 (7.9)	8 (3.4)	
Previous family violence					**0.004**
	No	155 (42.9)	68 (53.5)	87 (37.2)	
	Yes	206 (57.1)	59 (46.5)	147 (62.8)	
NPsychological violence					
Insulted me					≤ **0.001**
	No	388 (55.4)	341 (77.1)	47 (18.2)	
	Yes	**312 (44.6)**	101 (22.9)	211 (81.8)	
In the last 12 months					**0.007**
	No	209 (67.0)	57 (56.4)	152 (72.0)	
	Yes	103 (33.0)	44 (43.6)	59 (28.0)	
In the last 12 months					0.262
	Once	17 (16.5)	6 (13.6)	11 (18.6)	
	Few times	56 (54.4)	28 (63.6)	28 (47.5)	
	Many times	30 (29.1)	10 (22.7)	20 (33.9)	
Before the last 12 months					**0.014**
	Once	15 (14.6)	7 (15.9)	8 (13.6)	
	Few times	56 (54.4)	30 (68.2)	26 (44.1)	
	Many times	32 (31.1)	7 (15.9)	25 (42.4)	
Belittled or humiliated me					≤ **0.001**
	No	508 (72.6)	389 (88.0)	119 (46.1)	
	Yes	**192 (27.4)**	53 (12.0)	139 (53.9)	
In the last 12 months					**0.007**
	No	128 (66.7)	27 (50.9)	101 (72.7)	
	Yes	64 (33.3)	26 (49.1)	38 (27.3)	
In the last 12 months					0.052
	Once	11 (17.2)	8 (30.8)	3 (7.9)	
	Few times	39 (60.9)	14 (53.8)	25 (65.8)	
	Many times	14 (21.9)	4 (15.4)	10 (26.3)	
Before the last 12 months					**0.040**
	Once	9 (14.1)	6 (23.1)	3 (7.9)	
	Few times	35 (54.7)	16 (61.5)	19 (50.0)	
	Many times	20 (31.3)	4 (15.4)	16 (42.1)	
Tried to scare or intimidate me					≤ **0.001**
	No	515 (73.6)	404 (91.4)	111 (43.0)	
	Yes	**185 (26.4)**	38 (8.6)	147 (57.0)	
In the last 12 months					0.234
	No	138 (74.6)	25 (65.8)	113 (76.9)	
	Yes	47 (25.4)	13 (34.2)	34 (23.1)	
In the last 12 months					0.810
	Once	6 (12.8)	2 (15.4)	4 (11.8)	
	Few times	27 (57.4)	8 (61.5)	19 (55.9)	
	Many times	14 (29.8)	3 (23.1)	11 (32.4)	
Before the last 12 months					0.493
	Once	6 (12.8)	2 (15.4)	4 (11.8)	
	Few times	20 (42.6)	7 (53.8)	13 (38.2)	
	Many times	21 (44.7)	4 (30.8)	17 (50.0)	
Threatened to hurt me or someone I care					≤ **0.001**
	No	596 (85.1)	421 (95.2)	175 (67.8)	
	Yes	**104 (14.9)**	21 (4.8)	83 (32.2)	
In the last 12 months					0.267
	No	79 (76.0)	14 (66.7)	65 (78.3)	
	Yes	25 (24.0)	7 (33.3)	18 (21.7)	
In the last 12 months					0.800
	Once	4 (16.0)	1 (14.3)	3 (16.7)	
	Few times	16 (64.0)	4 (57.1)	12 (66.7)	
	Many times	5 (20.0)	2 (28.6)	3 (16.7)	
Before the last 12 months					0.800
	Once	5 (20.0)	2 (28.6)	3 (16.7)	
	Few times	12 (48.0)	3 (42.9)	9 (50.0)	
	Many times	8 (32.0)	2 (28.6)	6 (33.3)	
Physical violence					
Slapped me or threw something at me					≤ **0.001**
	No	599 (85.6)	428 (96.8)	171 (66.3)	
	Yes	**101 (14.4)**	14 (3.2)	87 (33.7)	
In the last 12 months					1.000
	No	89 (88.1)	12 (85.7)	77 (88.5)	
	Yes	12 (11.9)	2 (14.3)	10 (11.5)	
In the last 12 months					0.301
	Once	5 (41.7)	0 (0.0)	5 (50.0)	
	Few times	6 (50.0)	2 (100.0)	4 (40.0)	
	Many times	1 (8.3)	0 (0.0)	1 (10.0)	
Before the last 12 months					0.600
	Once	5 (41.7)	0 (0.0)	5 (50.0)	
	Few times	7 (58.3)	2 (100.0)	5 (50.0)	
	Many times	0 (0.0)	0 (0.0)	0 (0.0)	
Pushed or shoved me					≤ **0.001**
	No	577 (82.4)	419 (94.8)	158 (61.2)	
	Yes	**123 (17.6)**	23 (5.2)	100 (38.8)	
In the last 12 months					0.544
	No	104 (84.6)	18 (78.3)	86 (86.0)	
	Yes	19 (15.4)	5 (21.7)	14 (14.0)	
In the last 12 months					0.805
	Once	10 (52.6)	3 (60.0)	7 (50.0)	
	Few times	8 (42.1)	2 (40.0)	6 (42.9)	
	Many times	1 (5.3)	0 (0.0)	1 (7.1)	
Before the last 12 months					0.669
	Once	7 (36.8)	2 (40.0)	5 (35.7)	
	Few times	10 (52.6)	3 (60.0)	7 (50.0)	
	Many times	2 (10.5)	0 (0.0)	2 (14.3)	
Hit me with his/her fist or some object					≤ **0.001**
	No	647 (92.4)	439 (99.3)	208 (80.6)	
	Yes	**53 (7.6)**	3 (0.7)	50 (19.4)	
In the last 12 months					1.000
	No	52 (98.1)	3 (100.0)	49 (98.0)	
	Yes	1 (1.9)	0 (0.0)	1 (2.0)	
In the last 12 months					1.000
	Once	0 (0.0)	0 (0.0)	0 (0.0)	
	Few times	1 (100.0)	0 (0.0)	1 (100.0)	
	Many times	0 (0.0)	0 (0.0)	0 (0.0)	
Before the last 12 months					1.000
	Once	0 (0.0)	0 (0.0)	0 (0.0)	
	Few times	1 (100.0)	0 (0.0)	1 (100.0)	
	Many times	0 (0.0)	0 (0.0)	0 (0.0)	
Kicked or dragged me or beat me up					≤ **0.001**
	No	653 (93.3)	437 (98.9)	216 (83.7)	
	Yes	**47 (6.7)**	5 (1.1)	42 (16.3)	
In the last 12 months					1.000
	No	44 (93.6)	5 (100.0)	39 (92.9)	
	Yes	3 (6.4)	0 (0.0)	3 (7.1)	
In the last 12 months					1.000
	Once	3 (100.0)	0 (0.0)	3 (100.0)	
	Few times	0 (0.0)	0 (0.0)	0 (0.0)	
	Many times	0 (0.0)	0 (0.0)	0 (0.0)	
Before the last 12 months					1.000
	Once	3 (100.0)	0 (0.0)	3 (100.0)	
	Few times	0 (0.0)	0 (0.0)	0 (0.0)	
	Many times	0 (0.0)	0 (0.0)	0 (0.0)	
Choked me or burnt me on purpose					≤ **0.001**
	No	679 (97.0)	441 (99.8)	238 (92.2)	
	Yes	**21 (3.0)**	1 (0.2)	20 (7.8)	
In the last 12 months					1.000
	No	21 (100.0)	1 (100.0)	20 (100.0)	
	Yes	0 (0.0)	0 (0.0)	0 (0.0)	
In the last 12 months					1.000
	Once	0 (0.0)	0 (0.0)	0 (0.0)	
	Few times	0 (0.0)	0 (0.0)	0 (0.0)	
	Many times	0 (0.0)	0 (0.0)	0 (0.0)	
Before the last 12 months					1.000
	Once	0 (0.0)	0 (0.0)	0 (0.0)	
	Few times	0 (0.0)	0 (0.0)	0 (0.0)	
	Many times	0 (0.0)	0 (0.0)	0 (0.0)	
Hurt me with a knife, gun or other weapon					≤ **0.001**
	No	669 (95.6)	438 (99.1)	231 (89.5)	
	Yes	**31 (4.4)**	4 (0.9)	27 (10.5)	
In the last 12 months					1.000
	No	29 (93.5)	4 (100.0)	25 (92.6)	
	Yes	2 (6.5)	0 (0.0)	2 (7.4)	
In the last 12 months					1.000
	Once	1 (50.0)	0 (0.0)	1 (50.0)	
	Few times	0 (0.0)	0 (0.0)	0 (0.0)	
	Many times	1 (50.0)	0 (0.0)	1 (50.0)	
Before the last 12 months					1.000
	Once	1 (50.0)	0 (0.0)	1 (50.0)	
	Few times	1 (50.0)	0 (0.0)	0 (0.0)	
	Many times	0 (0.0)	0 (0.0)	1 (50.0)	
Sexual violence					
Demanded to have sexual intercourse					≤ **0.001**
	No	608 (86.9)	432 (97.7)	176 (68.2)	
	Yes	**92 (13.1)**	10 (2.3)	82 (31.8)	
In the last 12 months					0.250
	No	86 (93.5)	8 (80.0)	78 (95.1)	
	Yes	6 (6.5)	2 (20.0)	4 (4.9)	
In the last 12 months					0.472
	Once	1 (16.7)	0 (0.0)	1 (25.0)	
	Few times	4 (66.7)	2 (100.0)	2 (50.0)	
	Many times	1 (16.7)	0 (0.0)	1 (25.0)	
Before the last 12 months					0.759
	Once	0 (0.0)	0 (0.0)	0 (0.0)	
	Few times	4 (66.7)	2 (100.0)	2 (50.0)	
	Many times	2 (33.3)	0 (0.0)	2 (50.0)	
Forced me to have sex against my will					≤ **0.001**
	No	616 (88.0)	430 (97.3)	186 (72.1)	
	Yes	**84 (12.0)**	12 (2.7)	72 (27.9)	
In the last 12 months					0.149
	No	76 (90.5)	9 (75.0)	67 (93.1)	
	Yes	8 (9.5)	3 (25.0)	5 (6.9)	
In the last 12 months					1.000
	Once	0 (0.0)	0 (0.0)	0 (0.0)	
	Few times	5 (62.5)	2 (66.7)	3 (60.0)	
	Many times	3 (37.5)	1 (33.3)	2 (40.0)	
Before the last 12 months					0.144
	Once	0 (0.0)	0 (0.0)	0 (0.0)	
	Few times	4 (50.0)	3 (100.0)	1 (20.0)	
	Many times	4 (50.0)	0 (0.0)	4 (80.0)	
Forced me to do degrading sexual acts					≤ **0.001**
	No	653 (93.3)	439 (99.3)	214 (82.9)	
	Yes	**47 (6.7)**	3 (0.7)	44 (17.1)	
In the last 12 months					1.000
	No	45 (95.7)	3 (100.0)	42 (95.5)	
	Yes	2 (4.3)	0 (0.0)	2 (4.5)	
In the last 12 months					1.000
	Once	0 (0.0)	0 (0.0)	0 (0.0)	
	Few times	2 (100.0)	0 (0.0)	2 (100.0)	
	Many times	0 (0.0)	0 (0.0)	0 (0.0)	
Before the last 12 months					1.000
	Once	0 (0.0)	0 (0.0)	0 (0.0)	
	Few times	2 (100.0)	0 (0.0)	2 (100.0)	
	Many times	0 (0.0)	0 (0.0)	0 (0.0)	
VAW total score		1.00 [0.00 – 3.00]	0.00 [0.00 – 1.00]	**3.00 [2.00 – 6.00]**	≤ **0.001**
Psychological VAW total score		0.00 [0.00 – 2.00]	0.00 [0.00 – 1.00]	**2.00 [1.00 – 3.00]**	≤ **0.001**
Physical VAW total score		0.00 [0.00 – 0.00]	0.00 [0.00 – 0.00]	**0.00 [0.00 – 2.00]**	≤ **0.001**
Sexual VAW total score		0.00 [0.00 – 0.00]	0.00 [0.00 – 0.00]	**0.00 [0.00 – 2.00]**	≤ **0.001**

Data presented as absolute (n) and relative (%) frequencies. NV – group of women who reported not having suffered any violence. V – group of women who reported having suffered any violence. p – statistical significance index. Numbers highlighted in bold indicate association between categories.

* Chi-Squared test with adjusted residual analysis or Mann-Whitney test, when applicable. Significance set at 5% for all analysis

Some items from the instrument that measures VAW, in relation to quality of life and climacteric symptoms (CS-10) or female sexual function (FSFI-6), are presented in table 10. Within the premenopausal group, a positive relation was found between the VAW total score (ρ = 0.186, p = 0.047) or the psychological VAW total score (ρ = 0.194, p = 0.039), in relation to the CS-10 total score, illustrating that this type of violence, for this group, is related to a worsening of quality of life. Within the perimenopausal group, a positive relation was found between the physical VAW total score (ρ = 0.174, p = 0.013) or the sexual VAW total score (ρ = 0.163, p = 0.021), in relation to the CS-10 total score. All types of violence were positively related to the CS-10 of women in the postmenopausal group (Spearman's correlation, p ≤ 0.05 for all). On contrary, the sexual VAW total score was inversely related to both, the peri- (ρ = -0.152, p = 0.031) and the postmenopausal (ρ = -0.120, p = 0.019) groups, being related to an increase in sexual dysfunction in these groups. Furthermore, for all groups, higher CS-10 scores (more severe climacteric symptoms and lower quality of life) were related to a worsening in sexual function scores (Spearman's correlation, p ≤ 0.05 for all).

**Table 10:** Correlations among variables of interest

**A. CS-10**								
**Variable**	**Total (n = 700)**		**Premenopausal (n = 114)**		**Perimenopausal (n = 201)**		**Postmenopausal (n = 385)**	
	^ρ^	*p-value	^ρ^	*p-value	^ρ^	*p-value	^ρ^	*p-value
Have suffered any type of violence	**0.098**	**0.009**	0.091	0.334	0.077	0.280	**0.113**	**0.026**
VAW total score	**0.176**	≤ **0.0001**	**0.186**	**0.047**	0.133	0.059	**0.195**	≤ **0.0001**
Psychological VAW total score	**0.174**	≤ **0.0001**	**0.194**	**0.039**	0.124	0.080	**0.194**	≤ **0.0001**
Physical VAW total score	**0.160**	≤ **0.0001**	0.148	0.117	**0.175**	**0.013**	**0.158**	**0.002**
Sexual VAW total score	**0.129**	**0.001**	0.126	0.181	**0.163**	**0.021**	**0.116**	**0.023**
FSFI-6 total score	**-0.240**	≤ **0.0001**	**-0.195**	**0.038**	**-0.298**	≤ **0.0001**	**-0.253**	≤ **0.0001**
**B. FSFI-6**								
**Variable**		**Total (n = 700)**	**Premenopausal (n = 114)**		**Perimenopausal (n = 201)**		**Postmenopausal (n = 385)**	
	^ρ^	*p-value	^ρ^	*p-value	^ρ^	*p-value	^ρ^	*p-value
Have suffered any type of violence	**-0.077**	**0.041**	0.152	0.106	-0.024	0.734	**-0.140**	**0.006**
VAW total score	-0.052	0.173	0.100	0.289	-0.037	0.606	-0.088	0.085
Psychological VAW total score	-0.054	0.153	0.083	0.380	-0.016	0.824	-0.109	0.033
Physical VAW total score	0.032	0.402	0.164	0.082	0.027	0.703	0.012	0.813
Sexual VAW total score	**-0.112**	**0.003**	0.093	0.326	**-0.152**	**0.031**	**-0.120**	**0.019**
CS-10 total score	**-0.240**	≤ **0.0001**	**-0.195**	**0.038**	**-0.298**	≤ **0.0001**	**-0.253**	≤ **0.0001**

Spearman's correlations among the total scores of the 10-item Cervantes Scale (CS-10) or the 6-item Female Sexual Function Index (FSFI-6) with variables of interest regarding experienced violence, assessed by the World Health Organization (WHO) instrument of violence against women (VAW). Caption: ρ – Spearman correlation coefficient. p – statistical significance index. Significance set at 5% for all analyses. Numbers highlighted in bold indicate correlation between categories.

Consistent with this reasoning, when each group is dichotomized into V or NV any type of violence, the perception of being useless, measured by the CS-10 (Table 11), was aggravated in the peri- and the postmenopausal V groups (χ² ≤ 0.0001). Some symptoms, such as vaginal discomfort and/or dryness and skin dryness, were exhibited in higher proportion by the postmenopausal NV or V groups (χ² ≤ 0.0001). Similarly, all dimensions of the FSFI-6 instrument (Table 12), individually, indicate worse quality of female sexual function in all postmenopausal women NV or V (χ² ≤ 0.05 for all). In fact, considering the cutoff point proposed by the validated instrument for Brazilian climacteric women, 86.3% postmenopausal NV and 92.4% postmenopausal V presented sexual dysfunction (χ² ≤ 0.0001).

**Table 11:** 10-item Cervantes Scale (CS-10)

Variable		Total (N = 700)	Premenopausal (n = 114)		Perimenopausal (n = 201)		Postmenopausal (n = 385)		*p-value
			NV (n = 71)	V (n = 43)	NV (n = 131)	V (n = 70)	NV (n = 240)	V (n = 145)	
Hot flashes and/or night sweats									0.114
	0 (No symptom)	171 (24.4)	26 (36.6)	13 (30.2)	29 (22.1)	16 (22.9)	55 (22.9)	32 (22.1)	
	1	108 (15.4)	15 (21.1)	7 (16.3)	20 (15.3)	11 (15.7)	29 (12.1)	26 (17.9)	
	2	95 (13.6)	9 (12.7)	7 (16.3)	11 (8.4)	11 (15.7)	39 (16.3)	18 (12.4)	
	3	109 (15.6)	10 (14.1)	7 (16.3)	23 (17.6)	8 (11.4)	39 (16.3)	22 (15.2)	
	4	125 (17.9)	8 (11.3)	7 (16.3)	34 (26.0)	14 (20.0)	37 (15.4)	25 (17.2)	
	5 (Very severe)	92 (13.1)	3 (4.2)	2 (4.7)	14 (10.7)	10 (14.3)	41 (17.1)	22 (15.2)	
Heart beating quickly and out of control									0.201
	0 (No symptom)	274 (39.1)	30 (42.3)	16 (34.9)	41 (31.3)	23 (32.9)	114 (47.5)	51 (35.2)	
	1	120 (17.1)	11 (15.5)	10 (23.3)	22 (16.8)	14 (20.0)	30 (12.5)	33 (22.8)	
	2	98 (14.0)	7 (9.9)	4 (9.3)	25 (19.1)	12 (17.1)	35 (14.6)	15 (10.3)	
	3	96 (13.7)	10 (14.1)	7 (16.3)	24 (18.3)	10 (14.3)	23 (9.6)	22 (15.2)	
	4	73 (10.4)	9 (12.7)	3 (7.0)	14 (10.7)	8 (11.4)	22 (9.2)	17 (11.7)	
	5 (Very severe)	39 (5.6)	4 (5.6)	4 (9.3)	5 (3.8)	3 (4.3)	16 (6.7)	7 (4.8)	
Difficulty in sleeping									0.180
	0 (No symptom)	140 (20.0)	15 (21.1)	6 (14.0)	19 (14.5)	13 (18.6)	52 (21.7)	35 (24.1)	
	1	72 (10.3)	6 (8.5)	5 (11.6)	17 (13.0)	5 (7.1)	26 (10.8)	13 (9.0)	
	2	83 (11.9)	10 (14.1)	4 (9.3)	12 (9.2)	4 (5.7)	38 (15.8)	15 (10.3)	
	3	120 (17.1)	9 (12.7)	11 (25.6)	33 (25.2)	15 (21.4)	28 (11.7)	24 (16.6)	
	4	112 (16.0)	16 (22.5)	7 (16.3)	21 (16.0)	10 (14.3)	35 (14.6)	23 (15.9)	
	5 (Very severe)	173 (24.7)	15 (21.1)	10 (23.3)	29 (22.1)	23 (32.9)	61 (25.4)	35 (24.1)	
Aching in muscles and/or joints									0.239
	0 (No symptom)	107 (15.3)	12 (16.9)	3 (7.0)	15 (11.5)	12 (17.1)	45 (18.8)	20 (13.8)	
	1	99 (14.1)	14 (19.7)	5 (11.6)	18 (13.7)	10 (14.3)	29 (12.1)	23 (15.9)	
	2	88 (12.6)	12 (16.9)	5 (11.6)	24 (18.3)	5 (7.1)	28 (11.7)	14 (9.7)	
	3	108 (15.4)	9 (12.7)	11 (25.6)	21 (16.0)	9 (12.9)	41 (17.1)	17 (11.7)	
	4	113 (16.1)	7 (9.9)	9 (20.9)	21 (16.0)	9 (12.9)	42 (17.5)	25 (17.2)	
	5 (Very severe)	185 (26.4)	17 (23.9)	10 (23.3)	32 (24.4)	25 (35.7)	55 (22.9)	46 (31.7)	
Feeling a lack of energy									0.117
	0 (No symptom)	111 (15.9)	8 (11.3)	4 (9.3)	16 (12.2)	14 (20.0)	49 (20.4)	20 (13.8)	
	1	99 (14.1)	9 (12.7)	6 (14.0)	18 (13.7)	3 (4.3)	38 (15.8)	25 (17.2)	
	2	105 (15.0)	10 (14.1)	7 (16.3)	23 (17.6)	10 (14.3)	32 (13.3)	23 (15.9)	
	3	97 (13.9)	12 (16.9)	5 (11.6)	13 (9.9)	11 (15.7)	42 (17.5)	14 (9.7)	
	4	103 (14.7)	13 (18.3)	6 (14.0)	27 (20.6)	13 (18.6)	27 (11.3)	17 (11.7)	
	5 (Very severe)	185 (26.4)	19 (26.8)	15 (34.9)	34 (26.0)	19 (27.1)	52 (21.7)	46 (31.7)	
Perception of being useless									≤ **0.0001**
	0 (No symptom)	276 (39.4)	24 (33.8)	12 (27.9)	51 (38.9)	17 (24.3)	**121 (50.4)**	51 (35.2)	
	1	88 (12.6)	8 (11.3)	6 (14.0)	**25 (19.1)**	4 (5.7)	29 (12.1)	16 (11.0)	
	2	67 (9.6)	6 (8.5)	5 (11.6)	12 (9.2)	**14 (20.0)**	21 (8.8)	9 (6.2)	
	3	74 (10.6)	**13 (18.3)**	6 (14.0)	8 (6.1)	**13 (18.6)**	17 (7.1)	17 (11.7)	
	4	80 (11.4)	11 (15.5)	3 (7.0)	15 (11.5)	9 (12.9)	23 (9.6)	19 (13.1)	
	5 (Very severe)	115 (16.4)	9 (12.7)	11 (25.6)	20 (15.3)	13 (18.6)	29 (12.1)	**33 (22.8)**	
Feel anxious or nervous									**0.013**
	0 (No symptom)	97 (13.9)	10 (14.1)	2 (4.7)	16 (12.2)	7 (10.0)	**46 (19.2)**	16 (11.0)	
	1	120 (17.1)	11 (15.5)	4 (9.3)	24 (18.3)	10 (14.3)	**51 (21.3)**	20 (13.8)	
	2	106 (15.1)	4 (5.6)	9 (20.9)	**29 (22.1)**	8 (11.4)	31 (12.9)	25 (17.2)	
	3	114 (16.3)	13 (18.3)	7 (16.3)	21 (16.0)	11 (15.7)	42 (17.5)	20 (13.8)	
	4	108 (15.4)	16 (22.5)	6 (14.0)	18 (13.7)	13 (18.6)	31 (12.9)	24 (16.6)	
	5 (Very severe)	155 (22.1)	17 (23.9)	**15 (34.9)**	23 (17.6)	21 (30.0)	39 (16.3)	40 (27.6)	
Urine leaks									**0.002**
	0 (No symptom)	423 (60.4)	42 (59.2)	21 (48.8)	73 (55.7)	34 (48.6)	**159 (66.3)**	94 (64.8)	
	1	90 (12.9)	10 (14.1)	3 (7.0)	19 (14.5)	**17 (24.3)**	27 (11.3)	14 (9.7)	
	2	58 (8.3)	8 (11.3)	**9 (20.9)**	**17 (13.0)**	5 (7.1)	14 (5.8)	5 (3.4)	
	3	42 (6.0)	7 (9.9)	4 (9.3)	8 (6.1)	4 (5.7)	10 (4.2)	9 (6.2)	
	4	42 (6.0)	1 (1.4)	4 (9.3)	10 (7.6)	5 (7.1)	15 (6.3)	7 (4.8)	
	5 (Very severe)	45 (6.4)	3 (4.2)	2 (4.7)	4 (3.1)	5 (7.1)	15 (6.3)	**16 (11.0)**	
Vaginal discomfort and/or dryness									≤ **0.0001**
	0 (No symptom)	245 (35.0)	27 (38.0)	**22 (51.2)**	**57 (43.5)**	**34 (48.6)**	66 (27.5)	39 (26.9)	
	1	106 (15.1)	**17 (23.9)**	5 (11.6)	22 (16.8)	7 (10.0)	35 (14.6)	20 (13.8)	
	2	90 (12.9)	7 (9.9)	7 (16.3)	19 (14.5)	8 (11.4)	30 (12.5)	19 (13.1)	
	3	66 (9.4)	6 (8.5)	5 (11.6)	14 (10.7)	7 (10.0)	16 (6.7)	18 (12.4)	
	4	86 (12.3)	10 (14.1)	1 (2.3)	11 (8.4)	12 (17.1)	34 (14.2)	18 (12.4)	
	5 (Very severe)	107 (15.3)	4 (5.6)	3 (7.0)	8 (6.1)	2 (2.9)	**59 (24.6)**	**31 (21.4)**	
Skin dryness									≤ **0.0001**
	0 (No symptom)	82 (11.7)	**18 (25.4)**	**10 (23.3)**	16 (12.2)	7 (10.0)	23 (9.6)	8 (5.5)	
	1	103 (14.7)	11 (15.5)	3 (7.0)	20 (15.3)	9 (12.9)	36 (15.0)	24 (16.6)	
	2	118 (16.9)	12 (16.9)	6 (14.0)	26 (19.8)	14 (20.0)	39 (16.3)	21 (14.5)	
	3	118 (16.9)	8 (11.3)	11 (25.6)	24 (18.3)	17 (24.3)	36 (15.0)	22 (15.2)	
	4	122 (17.4)	12 (16.9)	7 (16.3)	29 (22.1)	13 (18.6)	40 (16.7)	21 (14.5)	
	5 (Very severe)	157 (22.4)	10 (14.1)	6 (14.0)	16 (12.2)	10 (14.3)	**66 (27.5)**	**49 (33.8)**	
CS-10 total score									0.400
	0 – 10	123 (17.6)	20 (28.2)	6 (14.0)	20 (15.3)	8 (11.4)	49 (20.4)	20 (13.8)	
	11 – 20	187 (26.7)	12 (16.9)	12 (27.9)	37 (28.2)	21 (30.0)	70 (29.2)	35 (24.1)	
	21 – 30	215 (30.7)	22 (31.0)	13 (30.2)	46 (35.1)	21 (30.0)	64 (26.7)	49 (33.8)	
	31 – 40	126 (18.0)	13 (18.3)	9 (20.9)	21 (16.0)	17 (24.3)	39 (16.3)	27 (18.6)	
	41 – 50	49 (7.0)	4 (5.6)	3 (7.0)	7 (5.3)	3 (4.3)	18 (7.5)	14 (9.7)	

Data presented as absolute (n) and relative (%) frequencies. p – statistical significance index. NV – group of women who reported not having suffered any violence. V – group of women who reported having suffered any violence. Violence measured by the World's Health Organization (WHO) instrument for violences against women (VAW). Numbers highlighted in bold indicate association between categories.

* Chi-Squared test with adjusted residual analysis. Significance set at 5% for all analysis

**Table 12:** 6-item Female Sexual Function Index (FSFI-6)

Variable		Total (N = 700)	Premenopausal (n = 114)		Perimenopausal (n = 201)		Postmenopausal (n = 385)		*p-value
			NV (n = 71)	V (n = 43)	NV (n = 131)	V (n = 70)	NV (n = 240)	V (n = 145)	
Desire									≤ **0.0001**
	Very low or none	177 (25.3)	10 (14.1)	8 (18.6)	24 (18.3)	13 (18.6)	**76 (31.7)**	**46 (31.7)**	
	Low	237 (33.9)	**35 (49.3)**	12 (27.9)	42 (32.1)	15 (21.4)	78 (32.5)	55 (37.9)	
	Moderate	186 (26.6)	18 (25.4)	13 (30.2)	33 (25.2)	23 (32.9)	67 (27.9)	32 (22.1)	
	High	80 (11.4)	6 (8.5)	8 (18.6)	**27 (20.6)**	**15 (21.4)**	14 (5.8)	10 (6.9)	
	Very high	20 (2.9)	2 (2.8)	2 (4.7)	5 (3.8)	4 (5.7)	5 (2.1)	2 (1.4)	
Arousal ^#^									≤ **0.001**
	Very low or none	71 (14.3)	6 (10.0)	6 (16.7)	6 (5.6)	6 (11.8)	**31 (18.8)**	16 (20.8)	
	Low	111 (22.4)	16 (26.7)	4 (11.1)	20 (18.7)	9 (17.6)	43 (26.1)	19 (24.7)	
	Moderate	187 (37.7)	26 (43.3)	9 (25.0)	46 (43.0)	15 (29.4)	61 (37.0)	30 (39.0)	
	High	95 (19.2)	10 (16.7)	**13 (36.1)**	26 (24.3)	13 (25.5)	23 (13.9)	10 (13.0)	
	Very high	32 (6.5)	2 (3.3)	4 (11.1)	9 (8.4)	**8 (15.7)**	7 (4.2)	2 (2.6)	
Lubrication ^#^									**0.001**
	Almost never or never	71 (14.3)	4 (6.8)	4 (11.1)	6 (5.7)	3 (5.8)	**35 (21.1)**	**19 (24.7)**	
	Few times	104 (21.0)	18 (30.5)	5 (13.9)	17 (16.0)	10 (19.2)	40 (24.1)	14 (18.2)	
	Sometimes	125 (25.2)	16 (27.1)	9 (25.0)	28 (26.4)	13 (25.0)	36 (21.7)	23 (29.9)	
	Mostly	128 (25.8)	14 (23.7)	12 (33.3)	**38 (35.8)**	14 (26.9)	33 (19.9)	17 (22.1)	
	Always or almost always	68 (13.7)	7 (11.9)	6 (16.7)	17 (16.0)	**12 (23.1)**	22 (13.3)	4 (5.2)	
Orgasm ^##^									**0.004**
	Almost never or never	49 (8.4)	6 (9.4)	1 (2.7)	2 (1.7)	4 (6.8)	20 (10.1)	**16 (14.5)**	
	Few times	89 (15.2)	5 (7.8)	3 (8.1)	13 (11.1)	6 (10.2)	38 (19.1)	**24 (21.8)**	
	Sometimes	148 (25.3)	20 (31.3)	10 (27.0)	37 (31.6)	9 (15.3)	50 (25.1)	22 (20.0)	
	Mostly	193 (32.9)	22 (34.4)	12 (32.4)	44 (37.6)	24 (40.7)	58 (29.1)	33 (30.0)	
	Always or almost always	107 (18.3)	11 (17.2)	11 (29.7)	21 (17.9)	16 (27.1)	33 (16.6)	15 (13.6)	
Satisfaction									**0.007**
	Very dissatisfied	159 (22.7)	12 (16.9)	6 (14.0)	18 (13.7)	11 (15.7)	**67 (27.9)**	**45 (31.0)**	
	Moderately dissatisfied	109 (15.6)	11 (15.5)	8 (18.6)	17 (13.0)	11 (15.7)	37 (15.4)	25 (17.2)	
	Neither satisfied nor dissatisfied	179 (25.6)	24 (33.8)	6 (14.0)	36 (27.5)	21 (30.0)	54 (22.5)	38 (26.2)	
	Moderately satisfied	175 (25.0)	19 (26.8)	15 (34.9)	41 (31.3)	15 (21.4)	58 (24.2)	27 (18.6)	
	Very satisfied	78 (11.1)	5 (7.0)	8 (18.6)	19 (14.5)	12 (17.1)	24 (10.0)	10 (6.9)	
Pain ^#^									≤ **0.0001**
	Always or almost always	78 (15.7)	6 (10.7)	2 (5.7)	6 (6.1)	4 (7.8)	**40 (23.5)**	**20 (23.5)**	
	Mostly	63 (12.7)	5 (8.9)	4 (11.4)	8 (8.1)	2 (3.9)	24 (14.1)	**20 (23.5)**	
	Sometimes	120 (24.2)	18 (32.1)	8 (22.9)	22 (22.2)	16 (31.4)	39 (22.9)	17 (20.0)	
	Few times	110 (22.2)	13 (23.2)	**14 (40.0)**	27 (27.3)	10 (19.6)	34 (20.0)	12 (14.1)	
	Almost never or never	125 (25.2)	14 (25.0)	7 (20.0)	**36 (36.4)**	19 (37.3)	33 (19.4)	16 (18.8)	
FSFI-6 total score									≤ **0.0001**
	≤ 21	574 (80.6)	59 (83.1)	26 (60.5)	91 (69.5)	47 (67.1)	**207 (86.3)**	**134 (92.4)**	
	≥ 22	136 (19.4)	12 (16.9)	**17 (39.5)**	**40 (30.5)**	**23 (32.9)**	33 (13.8)	11 (7.6)	

Data presented as absolute (n) and relative (%) frequencies. Legend: p – statistical significance index. NV – group of women who reported not having suffered any violence. V – group of women who reported having suffered any violence. Violence measured by the World's Health Organization (WHO) instrument for violences against women (VAW). Numbers highlighted in bold indicate association between categories.

* Chi-Squared test with adjusted residual analysis. Significance set at 5% for all analysis

## Discussion

To the best of our knowledge, this study represents the first attempt to assess the relationship between domestic violence and sexual function among climacteric women using the validated FSFI-6, alongside the evaluation of climacteric symptomatology and quality of life, as measured by the CS-10. Previous research on this topic has been limited, with most studies failing to utilize validated instruments to collect data on violence, sexuality, climacteric symptoms, and quality of life.^(7,23-29)^ In our study, domestic violence was associated with more severe climacteric symptoms, sexual function, and lower quality of life, especially in postmenopausal women.

In our sample of 700 women, we found that 38.8% experienced psychological violence, 34.9% experienced sexual violence, and 21.3% experienced physical violence. Women who reported experiencing violence were also found to be at greater risk for behaviors, tobacco and illicit drug use, and exhibited more severe climacteric symptoms and impaired sexual function. The NV group was primarily linked to psychological violence, while the V group was linked to all types of domestic violence investigated. This finding is supported by the higher scores in all domains and the overall score on the WHO VAW instrument for the V group. These results are consistent with prior studies highlighting the association between violence exposure and risk behaviors.^(30,31)^ Moreover, psychological violence, frequently underreported by both victims and witnesses, has historically been neglected.^(32)^ This was evident in our study, where participants in the NV group reported having experienced psychological violence.

Just over one-third of the sample (36.9%) reported experiencing violence at some point in their lives, consistent with WHO data.^(13)^ Postmenopausal women showed a higher prevalence of chronic diseases (42.3%), notably including back and neck pain, hypercholesterolemia, and type 2 diabetes (11.7%, 9.6%, 8.1%, respectively). However, these conditions did not show a significant correlation with violence. Previous research has indicated an increased risk of chronic diseases, including diabetes, and pain among those who have experienced violence.^(33)^

Our study identified associations between different types of violence and specific menopausal stages. Premenopausal women were more likely to report physical violence, while perimenopausal women were more frequently associated with sexual violence. Only one other study has also evaluated domestic violence across different menopausal stages, finding a connection between a history of sexual abuse and worsened health outcomes in climacteric women.^(23)^ Previous studies have examined the effects of violence at specific menopausal stages: premenopause,^(26)^ perimenopause,^(23,34)^ and postmenopause.^(23,26,28,34)^ Additionally, research on mid-aged women who experienced violence^(24,29)^ found poorer health outcomes in this group. A distinctive aspect of our study is its comprehensive evaluation of various types of domestic violence across different menopausal stages, using validated instruments to assess health issues specific to climacteric women. This research underscores the psychosomatic impact of violence on mid-aged women, revealing that it extends beyond being a mere public health issue confined to reproductive years.

Additionally, in our study, poorer quality of life was positively correlated with various forms of violence across all groups, with the postmenopausal group experiencing the most adverse outcomes. The perimenopausal women exhibited a positive correlation between poorer quality of life and climacteric symptoms with physical and sexual violence experiences, while the premenopausal group showed correlations with psychological violence and higher scores on the WHO VAW questionnaire. For postmenopausal participants, all forms of violence were correlated with reduced quality of life and worsened climacteric symptoms. Across the entire sample, a direct correlation was observed between worse symptomatology and quality of life with all violence variables and the total score on the WHO VAW questionnaire. This aligns with findings from Mendoza-Huertas et al.^(28)^ study involving 29 menopausal women who experienced violence, where these individuals exhibited poorer quality of life (higher CS-10 scores) and more severe climacteric symptoms compared to controls. Similarly, Moraes et al.^(7)^ found more severe climacteric symptomatology in a sample of 124 women aged 40 to 65 who experienced physical and sexual violence, with a higher prevalence of severe symptoms among those who experienced sexual or other forms of domestic violence. Schwarz et al.,^(26)^ using the modified Zerssen Symptom List (ZSL) instrument, found that menopausal women with higher ZSL scores, indicative of greater symptom severity, were associated with a history of physical, sexual abuse, and sexual harassment, independent of other variables.

In the context of our exploration, premenopausal women V were significantly associated with severe anxiety and nervousness (34.9%), while postmenopausal women V group exhibited very severe symptoms of feeling useless (22.8%). Moraes et al.^(7)^ study similarly reported a high prevalence (51.6%) of intense and moderate sadness/depression among their sample. Other researchers have underscored the impact of violence on the mental health of menopausal women. Uebelacker et al.^(27)^ found associations between verbal and physical abuse and symptoms of depression in postmenopausal women, while Stöckl et al.^(29)^ demonstrated that experiencing emotional abuse was linked to mild psychological issues such as concentration difficulties, fatigue, sleep disturbances, nervousness, depression, lack of motivation, and feeling overwhelmed, particularly in participants aged 50 to 65.

Throughout our study women who experienced violence also reported severe urinary leakage and very severe vaginal discomfort/dryness. Postmenopausal women NV group exhibited severe vaginal discomfort/dryness and dry skin. Gibson et al.^(25)^ research evaluated menopausal symptoms in women who experienced intimate partner violence, revealing associations between gynecological issues such as dyspareunia, vaginal dryness, and irritation with sexual violence. Night sweats were more prevalent in women who experienced emotional and physical violence compared to those who did not experience violence. Women subjected to emotional violence also reported more difficulty sleeping, night sweats, and dyspareunia. While our sample of perimenopausal and postmenopausal women experienced severe and very severe symptoms of hot flashes and/or night sweats, this was not associated with domestic violence.

Regarding sexual function, an inverse association was observed for all groups (NV or V); however, the postmenopausal group exhibited poorer sexual function correlated with any type of violence, whereas for the perimenopausal group, it was specifically correlated with sexual violence. As a part of our research, all menopausal groups exhibited an inverse correlation with sexual function, irrespective of experiencing violence. Specifically, the postmenopausal group showed an inverse correlation between good sexual function and any type of violence, sexual violence, and quality of life. Similarly, the perimenopausal group exhibited this correlation for sexual violence and quality of life, while the premenopausal group showed it only for the quality of life. Moraes et al.^(7)^ also assessed sexual function in their sample of menopausal women (n = 124), with 36 participants (29%) reporting and active sex life, of whom 20 (72.3%) considered it unsatisfactory. In our total sample, 22.7% reported unsatisfactory sexual function, with postmenopausal women reporting a higher frequency of this symptom (29.1%).

During our analysis, postmenopausal women V group were significantly associated with never or almost never having orgasms. Additionally, both postmenopausal women NV and V group were associated with very low sexual desire (31.7% in both groups), absence of lubrication (24.7% vs. 21.1%), low sexual satisfaction (31.0% vs. 27.9%), always or almost always experiencing pain during sexual intercourse (23.5% in both groups), and a worse total score for sexual dysfunction (2.4% vs. 86.3%). Postmenopausal NV were also associated with low or no arousal. The Seattle midlife Women's Health Study found a significant reduction in sexual desire in women during menopausal transition and early postmenopause, without an association with a history of sexual abuse.^(23)^ Conversely, other research has identified association between sexual, physical violence, and emotional abuse with pelvic problems, characterized by abdominal pain, pain or infections in intimate areas, and sexual problems, in women aged 50 – 65.^(29)^ Domestic violence has also been associated with symptoms of vaginal discharge, pain, and fatigue in middle-aged women.^(24)^

Our study has several limitations. Firstly, the cross-sectional design employed does not allow for precise assessment of causality in the relationships between variables. Additionally, our study was unable to assess economic and moral violence due to limitations of the validated instrument used. Despite our robust sample of 700 climacteric women, a significant number of missing data were observed, likely attributed to difficulties in interacting with the online questionnaire. Despite our efforts to provide instructional materials, notifications, and assistance to participants, as well as allowing ample time for questionnaire completion, these challenges persisted. Furthermore, our study sample represents only a specific region in the southern Brazil, and therefore, these results may not be generalizable to other populations.

This study investigated the prevalence and impact of violence across different stages of the climacteric, utilizing validated instruments (FSFI-6 and CS-10) for climacteric women in the southern region of Brazil. Our findings highlight a significant association between experiences of violence and deteriorations in sexual function, climacteric symptoms, and quality of life, contributing uniquely to the existing literature into this specific population. When compared with the findings of WHO reports and other literature, the prevalence of violence observed in our study reveals both similarities and key differences. For instance, the higher incidence of psychological violence in our sample aligns with global data, while variations in the types of violence observed may be attributed to cultural and regional factors specific to our study setting.

## Conclusion

These findings carry significant implications for clinical practice, underscoring the multifaceted ways in which violence affects women's health. Addressing violence, often a taboo subject in society, is crucial for promoting the health and well-being of women, particularly during the menopausal transition. Effective public policies aimed at safeguarding women across the lifespan and enhancing the quality of life for menopausal women, who constitute a substantial proportion of the elderly population in Brazil and beyond, are imperative.
